# A topological classifier to characterize brain states: When shape matters more than variance

**DOI:** 10.1371/journal.pone.0292049

**Published:** 2023-10-02

**Authors:** Aina Ferrà, Gloria Cecchini, Fritz-Pere Nobbe Fisas, Carles Casacuberta, Ignasi Cos

**Affiliations:** 1 Departament de Matemàtiques i Informàtica, Universitat de Barcelona, Barcelona, Catalonia, Spain; 2 Institut de Matemàtica de la Universitat de Barcelona (IMUB), Barcelona, Spin; 3 Serra-Húnter Fellow Programme, Barcelona, Catalonia, Spain; Naval Postgraduate School, UNITED STATES

## Abstract

Despite the remarkable accuracies attained by machine learning classifiers to separate complex datasets in a supervised fashion, most of their operation falls short to provide an informed intuition about the structure of data, and, what is more important, about the phenomena being characterized by the given datasets. By contrast, topological data analysis (TDA) is devoted to study the shape of data clouds by means of persistence descriptors and provides a quantitative characterization of specific topological features of the dataset under scrutiny. Here we introduce a novel TDA-based classifier that works on the principle of assessing quantifiable changes on topological metrics caused by the addition of new input to a subset of data. We used this classifier with a high-dimensional electro-encephalographic (EEG) dataset recorded from eleven participants during a previous decision-making experiment in which three motivational states were induced through a manipulation of social pressure. We calculated silhouettes from persistence diagrams associated with each motivated state with a ready-made band-pass filtered version of these signals, and classified unlabeled signals according to their impact on each reference silhouette. Our results show that in addition to providing accuracies within the range of those of a nearest neighbour classifier, the TDA classifier provides formal intuition of the structure of the dataset as well as an estimate of its intrinsic dimension. Towards this end, we incorporated variance-based dimensionality reduction methods to our dataset and found that in most cases the accuracy of our TDA classifier remains essentially invariant beyond a certain dimension.

## Introduction

The ability to capture detailed high-dimensional statistics of large datasets has earned deep learning the reputation of general solver for a wide range of complex problems [1–3]; from image analysis [4, 5] to object recognition [6], climate prediction [7, 8], genomic analyses and behavioural prediction [9] or hidden variable identification for brain dynamics [3]. However, the downside of deep learning, as of any black-box AI technique, is the poor explainability of results and of the structure of the datasets, which necessarily bounds interpretability [10–12]. Possibly more puzzling, deep learning commits rare but unpredictable clustering errors, such as confusing a lion with a library, or a lamp with a traffic light [13]. While odd, it is precisely the lack of a principled explanation leading to such failures that makes of deep learning a technique with significant trust issues, in particular for AI in life-threatening decision-making scenarios [14, 15].

By contrast, topology is a branch of mathematics devoted to characterize the structure of high-dimensional datasets by formal means. In brief, if a dataset can be represented as a point cloud in a hyperspace, topological data analysis (TDA) may characterize its connectivity, or cycles within the cloud, or other shape features, by means of a set of metric descriptors that encompass all dimensions. Therefore, although not conceived for problem solving or to capture data variability per se, if one applies TDA to datasets of different classes within a classification problem, it will yield descriptors specific to each class. Consistently with this, some studies have proposed the use of TDA metrics as a preliminary stage to extract features for machine learning classifiers, e.g., for medical image analysis [16–18], chemical components [19, 20], or computer science problems [21]. Such classification problems do not operate in a multidimensional dataset or in a subsequent reduced dimensionality space [22, 23], but rather in the domain of meaningful topological feature vectors [24–26], with a consequent interpretability gain.

Topological descriptors, such as persistence landscapes [27, 28] or persistence-based Gaussian kernels [29], have proved to be powerful tools for statistical analyses. While using such descriptors to improve machine learning classifiers is a promising research avenue that facilitates tracing class specificities back to the structure of data, our goal in this work is to use instead TDA as a classifier by itself. In brief, if different subsets of data—belonging to different classes—yield different topological descriptors, it is plausible that such differences across descriptors can be directly implemented for classification purposes.

In this article, we introduce a classifier inspired on this principle. The bonus of such a classifier with respect to a classical machine learning one is that it should provide an informed intuition of the specific aspects of the dataset responsible for separability of classes. The same principle was used in [30] to obtain a new TDA-based semi-supervised learning method. A different mechanism for TDA-based classification was described in [31, 32] as a refinement of nearest neighbour classifiers by exploiting the local structure of Vietoris–Rips simplicial complexes associated with a data cloud.

As a testbed for our classifier, we used a challenging classification problem in dire need of explainability, which is the state cortical brain network during performance of specific tasks [33–35]. In particular, we focused on the characterization of the brain network of motivation, as defined in the context of a decision-making task between precision reaches [36] by different levels of social pressure.

Reward and motivation are two fundamental drives of human behaviour. Consistently with this, a large number of studies in neuroscience have intended a careful identification and characterization of the brain centers of reward processing, most often based on analyses of functional magnetic resonance imaging (fMRI) recordings in humans. However, one of the questions that remains to be fully answered is how the different expressions of motivation are distributed across the brain network.

We analysed a set of electro-encephalograms (EEG) recorded as the decisions unfolded from eleven participants, by turning the problem into a three-class classification problem, in which we aimed at explaining the differences across these states on the grounds of our TDA classifier. This yielded two main results: first, the TDA classifier obtained accuracies comparable to those obtained by a nearest neighbour classifier; second, accuracy strongly depended on the shape of data—since TDA operates on simplicial complexes built from datasets—but not on the amount of explained variance achieved by a dimension-reducing projection. In summary, specific topological descriptors indeed provide reliable ensemble characterizations of high-dimension neural states, and yield an avenue for data explainability complementary to machine learning.

## Materials and methods

The main purpose of this work was to introduce a novel classifier based on topological principles, as well as the necessary assessment and characterization of its performance. We therefore required a rich enough dataset, which had been previously tested by traditional machine learning classifiers, so as to establish a baseline of performance that enabled an informed comparison. Because of this, we chose a previously recorded dataset, which is available at https://www.kaggle.com/ds/3333171, whose specifics are described in the next subsection. The reader may refer to the original neuroscience study [36] for a more thorough description. In addition to providing an advantageous baseline to fall back on to validate our classifier, we chose this dataset in order to study the underlying structure and latent dimensionality of electro-encephalographic recordings by means of topological data analysis.

### Brain states of motivation

The dataset used to test our topological classifier was collected during a previous study aimed at the characterization of the influence of social pressure on movement decisions and on the choice of movement parameters, both at the behavioural and cortical level [36]. Because of this, arm kinematics and high-density electro-encephalograms (EEG) were recorded. Specifically, each participant was instructed to choose one of two targets and to perform a precision reach towards the target of choice. To be able to perform a controlled manipulation of the participant’s motivation, and to obtain reliable measures of its influence at a behavioural and neural level, each of the two sessions was divided into six blocks of trials, each composed of 108 trials. Each block was performed under one of three levels of social pressure. The order of the blocks was counterbalanced across participants.

The manipulation of the participant’s motivation was implemented by the presence or absence of one of several simulated (virtual) partners. In the first (baseline or Solo condition), the participant performed alone. At the end of each trial, the participant was informed of the precision attained during the previous reach with a horizontal green bar ranging from 0 to 100%. In the second (Easy) and third (Hard) condition, the participant was informed that a virtual partner (invisible to the participant) would perform alongside. At the end of each trial, the participant was informed of his/her precision and of that of the virtual partner. The manipulation was such that the average aiming accuracy of the partner during the Easy condition was lower than that of the participant, while it was higher at the Hard condition. The level of social pressure remained constant throughout each block, maintaining the same virtual partner. The goal of this manipulation was to induce an implicit bias that motivated participants to adapt their aiming performance. To reinforce the implicit nature of this manipulation, participant were instructed not to compete and to focus on their own performance while disregarding the partner.

The six blocks of trials per session were organized in groups of three. Each group consisted of one Solo, one Easy and one Hard blocks of trials. The goal of this manipulation was to induce three distinct motivated states as a function of the level of social pressure exerted.

### Structure of the dataset

A full description of the data conditioning process can be found in the original study [36]. A summary description is provided next. The dataset consisted of EEG fragments recorded from each participant while they performed the Solo, Easy and Hard blocks of trials. Since the study focused on assessing the baseline changes of cortical brain state as a function of experimental condition, a 1200 ms interval was chosen during the beginning of each trial. It started 800 ms before the first stimulus onset—the initial cue—and ended 400 ms after (see Fig 5A), before any movement or stimuli were presented on the screen at that trial.

In brief, the 1200 ms time series resulting from each channel and trial was filtered with a 4th order notch filter at 50, 100 and 150 Hz to remove noise originating from the power supply line. Furthermore, as customary for scalp EEG recordings, the time series frequency band was constrained between 0.1 and 100 Hz by means of a 4th order band-pass Butterworth filter. Electrodes with EEG level exceeding either 200 V or voltage step/sampling 50 V within intervals of 200 ms were removed from further analysis. Baseline was corrected by removing the overall mean potential across electrodes, and the datasets were *z*-scored using the recordings during trial block types 1 and 2 of each session—when the participant was playing solo, as a baseline reference.

Eye-related artefacts were removed by means of independent component analysis (ICA), implemented with a custom-made open-source toolbox (www.fieldtrip.com) and EEGLAB scripts (sccn.ucsd.edu/eeglab, UC San Diego, CA, USA). The procedure to identify eye-movement related sources was semi-automatized, first correlating each source obtained with the signal from the electrodes recording eye movements to obtain a first metric of relatedness. Second, all sources were visually inspected to corroborate that their shape and spatial location matched those of ocular artefacts. Eye-related sources were removed and the cleaned signal obtained by inverting the ICA process. A final source space was obtained by applying ICA again, but forcing the resulting space to be of smaller dimension than the electrode space, in order to capture a few independent areas of the brain whose signals are sent to the electrodes. Thus the dimension of the source space may be different for each participant—usually less than fifty [37].

The computation of the projection of the data on the source space was performed with custom-made MATLAB scripts based on the EEGLAB library, combined with the electrode spatial location map. The Brain Products Unicap 64 configuration (Brain Products GmbH, Gilching, Germany) was used to establish a spatial reference between the electrode placement and to perform source localization. A spherical head model was assumed. ICA projections serve a dual purpose: first, to distribute the information contained in the electrode signals along directions of maximal inter-independence between dimensions; second, to provide a rough anatomical estimate for the location of the brain sources generating the signals recorded by the electrodes, on the brain cortical surface.

In summary, the original dataset consisted, per trial, of a variable number of channels (electrodes or sources), denoted as *N*_*C*_, lasting 1200 ms each. Each participant performed 12 blocks of 108 trials. Blocks were recorded during two different sessions, containing 6 blocks each, balancing out the number of motivated blocks.

Ultimately, the time series were averaged across the 1200 ms, yielding a mean characterization of the three brain states characterized (Solo, Easy and Hard).

### Persistence descriptors

Topological data analysis is a branch of mathematics based on algebraic topology aiming to detect and represent structural features of datasets, such as sparseness, flares or cycles. Its main tool is persistent homology [38–42], an algebraic characteristic of simplicial complexes equipped with a real-valued filtering function. Persistent homology is well suited for describing the shape of a point cloud along a range of resolution scales.

Formally, a point cloud is a finite subset $X\subset R^{d}$ for some *d* ≥ 1 viewed as a metric space by means of the Euclidean distance. The *Vietoris–Rips filtration* [43, 44] associated with *X* is a nested family of abstract simplicial complexes *V*_*t*_(*X*) for *t* ≥ 0, where *V*_*t*_(*X*) has a 0-simplex (that is, a vertex) for each point in *X* and a *k*-simplex with *k* ≥ 1 for each collection of points *v*_0_, …, *v*_*k*_ in *X* such that ‖*v*_*i*_ − *v*_*j*_‖ ≤ *t* for all *i*, *j*.

The inclusions *V*_*s*_(*X*) ⊆ *V*_*t*_(*X*) for *s* ≤ *t* induce morphisms *H*_*n*_(*V*_*s*_(*X*)) → *H*_*n*_(*V*_*t*_(*X*)) for *n* ≥ 0, where *H*_*n*_ denotes *n*-dimensional simplicial homology [45]. We use the GUDHI Python Library [46] for calculations. Although any coefficient field is suitable to compute persistent homology [47], finite fields of prime order *p* are commonly used. In GUDHI, the largest possible choice is *p* = 46337 and the default choice is *p* = 11, which is used in the present work.

The *birth parameter* *b* of a homology generator in dimension *n* is the smallest value of *t* such that *H*_*n*_(*V*_*t*_(*X*)) contains the given generator, and the *death parameter* *d* is the smallest value of *t* where that generator is mapped to zero. The *persistence* or *lifetime* of a homology generator is the difference *d* − *b*.

The *persistence diagram* associated with the Vietoris–Rips filtration of *X* in homological dimension *n* consists of all birth-death pairs (*b*, *d*) for a basis of *n*-dimensional homology generators, drawn above the diagonal *y* = *x* of the first quadrant in $R^{2}$. Points that are close to the diagonal (i.e., with a short lifetime) may correspond to inessential phenomena, while those with large lifetimes reflect persistent shape features of the given dataset. In some cases, however, the distribution of points near the diagonal carries relevant information that should not be neglected.

Persistence diagrams in dimension 0 contain information about connected components of Vietoris–Rips complexes, specifically about the way in which connected components merge as the parameter *t* increases. Persistence diagrams in dimension 1 depict the appearance and disappearance of 1-cycles, while persistence diagrams in dimensions *n* ≥ 2 represent the evolution of *n*-dimensional cavities in the Vietoris–Rips complexes.

The *total persistence* of a persistence diagram is defined as Σ_*i*_(*d*_*i*_ − *b*_*i*_), where the sum is taken over all points with finite persistence in the diagram; *b*_*i*_ denotes the birth parameter of the *i*th point and *d*_*i*_ is the corresponding death parameter.

Two fundamental results endow persistent homology with the robustness required for a rigorous mathematical theory with real-world applications. The first one is the fact that persistence diagrams are well-defined [39], that is, do not depend on the choice of a basis of homology generators. The second one is *stability* [48, 49], i.e., small perturbations in the data can only yield minor perturbations in the corresponding persistence diagrams.

**Fig 1:**
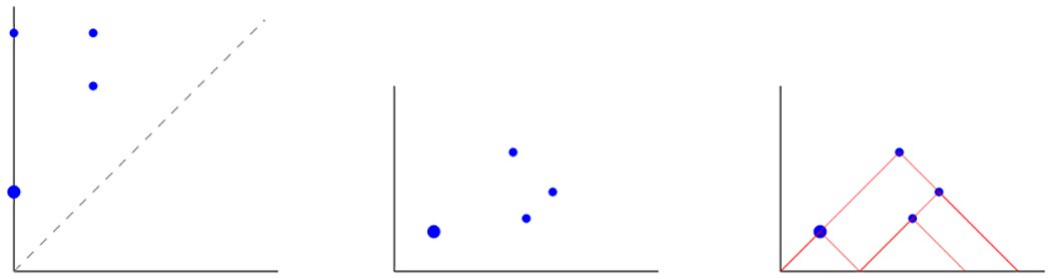
Landscape. Persistence landscape (right) obtained from a persistence diagram (left) by means of a 45° rotation and rescaling (middle). Dot size indicates multiplicity.

Dissimilarity between persistence diagrams can be measured by the *bottleneck distance*, to which the stability theorem refers when claiming that two diagrams are close to each other [38]. However, persistence diagrams equipped with the bottleneck distance are not well-suited for statistical analyses, since it is not feasible to compute averages of persistence diagrams [50]. For this purpose, a convenient summary of a persistence diagram is its *landscape* [27, 28]. A persistence landscape is a sequence of piecewise linear functions obtained by rotating the diagram 45 degrees clockwise (Fig 1) and choosing the *k*-highest point for each *k* ≥ 1 in the resulting figure.

**Fig 2:**
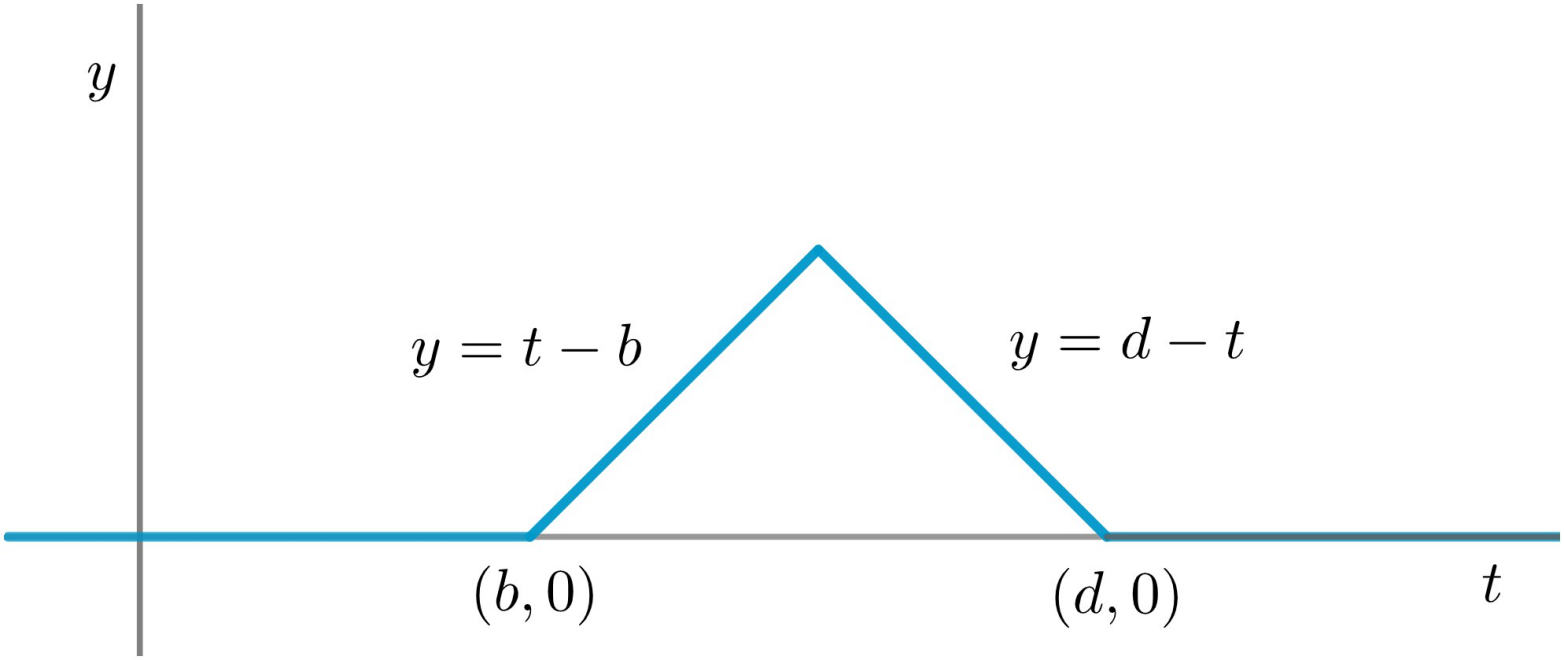
Tent function with birth parameter *b* and death parameter *d*.

More precisely, for each point (*b*, *d*) in a given persistence diagram, one considers a *tent function* Λ_(*b*,*d*)_ (*t*) = max{0, min{*t* − *b*, *d* − *t*}}, as depicted in Fig 2, and defines $\lambda_{k}:R\to R$ for each *k* ≥ 1 as $\lambda_{k}\left(t\right)=\text{kmax}\left\{\Lambda_{\left(b_{i},d_{i}\right)}\left(t\right)\right\}$, where {(*b*_*i*_, *d*_*i*_)} is the set of all points in the given persistence diagram and kmax returns the *k*-th largest value of a given set of numbers, or zero if there is no *k*-th largest value. Consequently, λ_*k*_ = 0 for sufficiently large values of *k*. The first landscape levels λ_1_, λ_2_ … represent the most significant features from the persistence diagram, while the last ones correspond to points near the diagonal and hence ephimerous phenomena.

Despite their usefulness, persistence landscapes are memory-wise expensive when handling large datasets. To overcome this problem, *silhouettes* were introduced in [51] by considering a weighted average of the tent functions used to build a landscape:

$$\phi_{w}\left(t\right)=\frac{\sum_{i=1}^{m}\;w_{i}\;\Lambda_{\left(b_{i},d_{i}\right)}\left(t\right)}{\sum_{i=1}^{m}w_{i}}.$$

In this work we chose lifetimes *w*_*i*_ = *d*_*i*_ − *b*_*i*_ as weights and used the resulting silhouettes as persistence summaries for our analyses. Since lifetimes need to be finite, points with infinite persistence were discarded.

For the classification purposes in this article, we only used persistence diagrams in homological dimension zero. Higher dimensions were not considered in the study since, generically, no significant variation of persistence summaries can be expected by adding a single point to a training dataset, except possibly in dimension zero. This implies that *b*_*i*_ = 0 for all *i*, and therefore lifetimes coincide with death values, while *w*_*i*_ = *d*_*i*_. Thus a silhouette represents density of points in the corresponding persistence diagram for each interval of persistence values, yet weighted by lifetime.

We measure dissimilarity between persistence diagrams by discretizing silhouettes into vectors of 1000 components and computing Euclidean distances between such vectors.

### Topological classifier

A typical classification process starts with partitioning a labeled dataset *D* into training and testing sets, assuming knowledge of the label class *c* to which each input *x* belongs in the training set. Accuracy is defined as the percentage of correctly classified datapoints from the testing set.

Thus, if we have a dataset *D* containing *m* training datapoints *D* = {(*x*_*i*_, *c*_*i*_)} with *i* = 1, …, *m*, the purpose of classifying is, for any given point *x*, to return its best predicted class *c*(*x*). Therefore, a critical question for a classifier algorithm is to define an appropriate metric of similarity between datapoints, so that similar points belong the same class. Typically, given two points *x* and *y*, similarity is quantified by a distance function *d*(*x*, *y*) between them. For example, the *nearest neighbour algorithm* uses the Euclidean distance. Given a datapoint *x* from the testing dataset, and given the training data {(*x*_*i*_, *c*_*i*_)}, the nearest neighbour algorithm classifies *x* as follows:

1. Calculate the Euclidean distance *d*_*i*_ = *d*(*x*, *x*_*i*_) of the point *x* to each of the training points *x*_*i*_.
2. Find a point $x_{i_{*}}$ in the training dataset such that $d_{i_{*}}={\text{min}}_{i}\;\left\{d_{i}\right\}$.
3. Assign the class label $c\left(x\right)=c_{i_{*}}$. If there are equidistant points with different labels, the algorithm selects the class containing the largest number of points.

Our persistence-based topological classifier follows instead the next procedure:

1. The training set is split into classes according to given labels.
2. For each class label *c* in the training set, calculate the corresponding persistence silhouette *S*_*c*_ in homological dimension 0 with lifetimes as weights.
3. To classify an input *x* from the testing set, add *x* to the cloud of training datapoints *X*_*c*_ of each class label *c*. Then, recompute the persistence silhouettes *S*_*c*,*x*_ for the datasets *X*_*c*_ ∪ {*x*}, and finally calculate the Euclidean distance *d*(*S*_*c*_, *S*_*c*,*x*_) between the newly obtained silhouettes and the former ones.
4. Assign the class label *c*(*x*) = c_*_ where c_*_ = argmin_*c*_{*d*(*S*_*c*_, *S*_*c*, *x*_)}.

**Fig 3:**
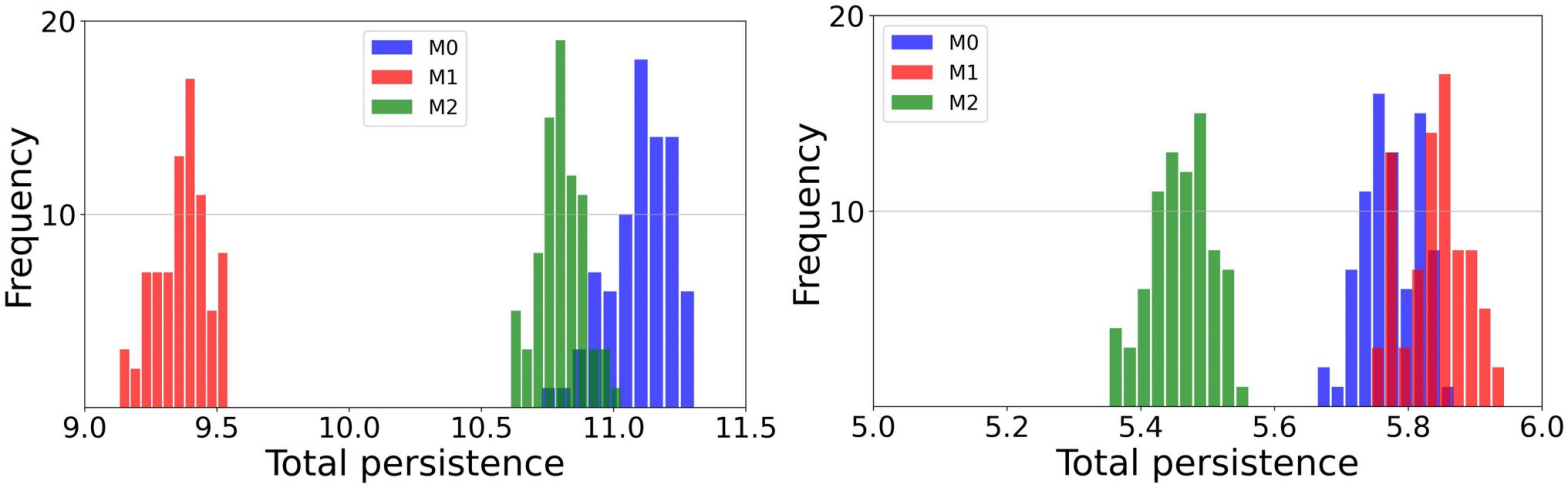
Total persistence by motivational states. Distribution of total persistence Σ_*i*_(*d*_*i*_ − *b*_*i*_) in dimension zero of participant 1 (left) and participant 8 (right) of the point clouds corresponding to three motivational states: *M*_0_ blue, *M*_1_ red, *M*_2_ green. Numbers in the *x*-axis are total persistence values, whose range varies depending on the recordings of each participant.

The underlying assumption is that point clouds sampled from different classes exhibit recognizably different shapes, as suggested by S2 Fig. The plausibility of this claim was tested by means of bootstrapping on each motivational state by repeatedly sampling 75% of each data cloud randomly with replacement 80 times. The resulting distributions of zero-dimensional total persistence are shown in Fig 3 for participants 1 and 8. The statistical null hypothesis that the distributions were pairwise equal was rejected for all participants by means of a Kolmogorov–Smirnov test with *p*-values below 0.0001.

**Fig 4:**
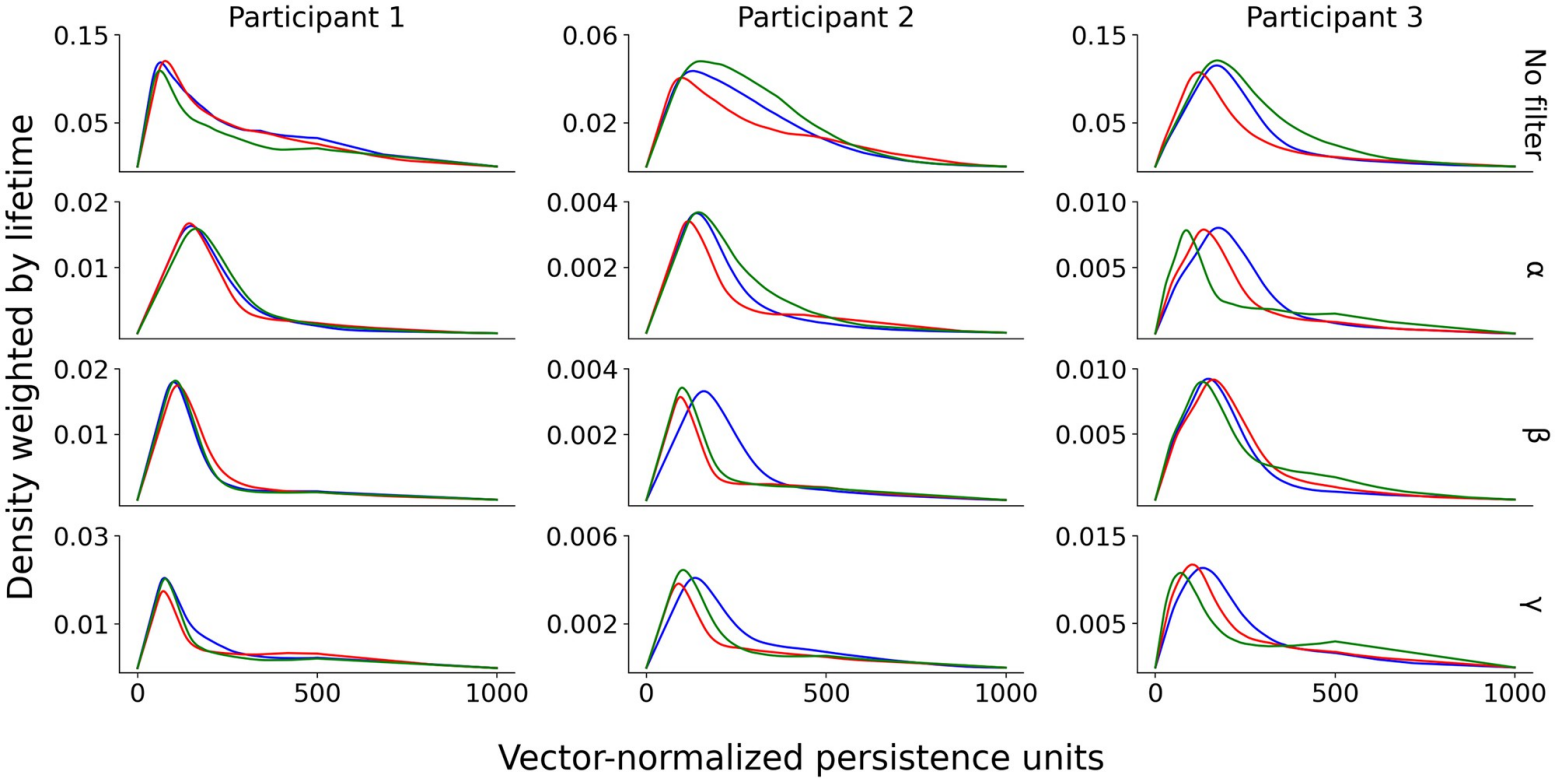
Persistence silhouettes. Silhouettes from persistence diagrams in dimension zero for each motivational state (*M*_0_: blue, *M*_1_: red, *M*_2_: green) for each frequency band (*α*, *β*, *γ*) plus the unfiltered dataset (no filter) for participants 1 to 3 in the space of sources without dimensionality reduction. The *x*-axis represents lifetime lengths of connected components of the Vietoris–Rips complex, normalized into 1000 vector units by equating ranges. The *y*-axis represents density of points in the persistence diagram weighted by their lifetime, uniformized by equating height in the pictures.

Further evidence of the difference between persistence descriptors of the three motivational states was obtained by drawing silhouettes in dimension zero for each state, as depicted in Fig 4 for participants 1 to 3, separated by frequency bands.

If an unlabeled point is added to the point cloud of its own class, the resulting topology should not fundamentally change. By contrast, if a point is added to a point cloud of a different class, then the topology should be altered more visibly.

### Analysis pipeline

**Fig 5:**
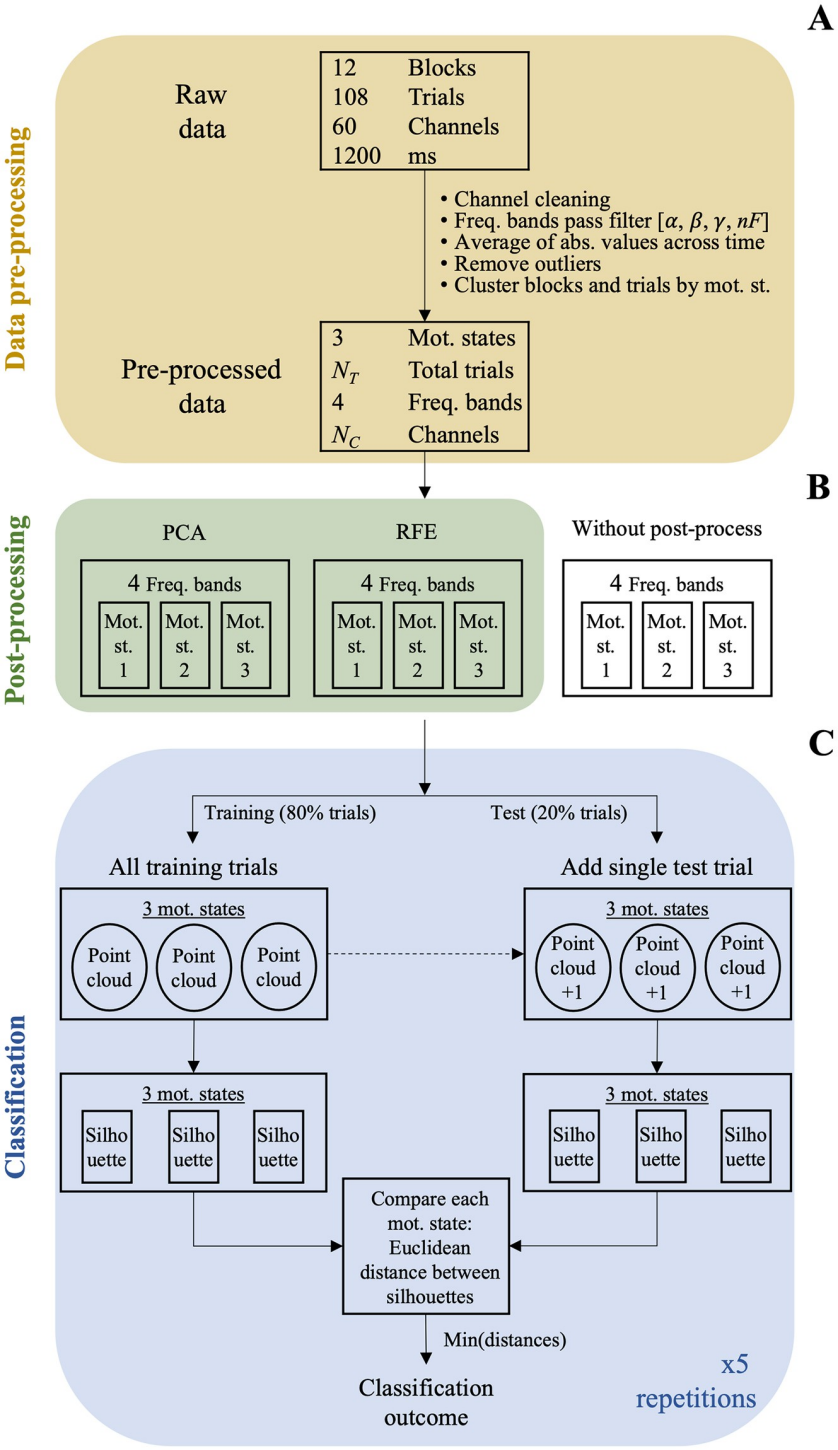
**A: Pre-processing schematic**. Channels are either electrodes or sources. Each channel of raw data was band-pass filtered into the three typical EEG bands (*α*: low; *β*: medium; *γ*: high). Channels containing artifacts or noisy information were removed, resulting in a final number of *N*_*C*_ channels. Absolute values of time series were averaged for each channel. Outliers were removed from the resulting dataset. **B: Post-processing schematic**. Principal component analysis (PCA) or recursive feature elimination (RFE) were used. No post-processing was performed on raw data. **C: Classification schematic**. 80% of the dataset was used for training purposes and 20% for testing ones. Persistence silhouettes were used for classification. Abbreviations in the image are freq.: frequency; abs.: absolute; mot.: motivational; st.: state.

For each of the eleven participants in the study (four male and seven female aged 55±5.8), the dataset consisted of 1200 ms × 60 electrodes EEG segments, repeated over 12 blocks (six per session) of 108 trials each (Fig 5A). The level of social pressure leading to a specific motivated state was maintained constant across each block, and there were four blocks for each motivated state (two in each session).

#### Pre-processing

The data used in the present article was provided both in electrode and source space directly, in three frequency bands of interest. A full description of the data processing is available in the original study [36]. However, a summary description is provided here for completeness and for notational purposes.

- First, data were visually inspected to identify and remove noisy or artefactual channels from further analysis. The number of channels surviving this process is denoted by *N*_*C*_.
- Second, EEG signals encompass a spectrum ranging 0.01–100 Hz, which were distributed into three typical frequency bands: *α* (8–15 Hz), *β* (15–32 Hz), *γ* (32–80 Hz), obtaining three band-passed versions of the original temporal series. Each of these signals was analysed in the same fashion, and independently of each other, alongside with an unfiltered baseline version of the original signal. This distribution of frequency bands responds to the established association of brain function with power fluctuations in specific bands and electrode locations. Band-pass filtering was performed with custom-made scripts in Python, using the iirfilter() function from the scipy.signal library [52]. Previous analyses on previous studies suggest that motivation-related modulations belong in the high-gamma band, thus suggesting that motivation related biases should be better encoded in the higher frequency band [36].
- Third, each electrode or source absolute value was averaged across the 1200 ms window of observation, obtaining a dataset organised as a matrix of *N*_*T*_ trials by *N*_*C*_ channels. Outliers exceeding twice the standard deviation from the average of norms of datapoints were disregarded from further analysis.

Classification of brain states was conducted within the signals obtained for each band-pass independently, as well as with an unfiltered version of the original signal.

#### Post-processing

Two different dimensionality reduction methods were used to the pre-processed dataset by means of the sklearn library [53], yielding dimensions between 2 and 10.

- Principal component analysis (PCA) is a linear projection onto a lower-dimensional space of principal components, where the first principal component of a point cloud is the one that explains the most variance, and each successive principal component explains the most variance in what is left once the effect of the previous components is removed [22].
- Recursive feature elimination (RFE) consists of successively removing coordinates with the lowest impact on the accuracy of a classifier [54]. In our study, RFE was applied to the pre-processed dataset using a logistic regression model to assign weights to the features.

#### Classification

Once each dataset (for each frequency band) was properly formatted, it was input into our TDA classifier (Fig 5C). From this moment on, trials were considered as points of a data cloud to be classified. The classification operation was carried out as described in the topological classifier subsection. Each classification was repeated 5 times and the resulting accuracies were averaged over the 5 repetitions. At each repetition, post-processed datasets were partitioned into 80% training data and 20% testing data. In the case of raw data (no dimensionality reduction), pre-processed datasets were used.

The analyses were performed with the original signals in electrode space as well as with their projections onto the brain source space.

## Results

To first establish a baseline of accuracy that would enable an assessment of the influence of dimensionality reduction techniques on the classificacion process, we tested our DA classifier on the original EEG signals projected on source space; see Analysis pipeline. Second, we tested the influence of two dimensionality reduction methods (PCA and RFE) on the classification process. Third, to further assess the influence of the ICA projection on the classifier, we also tested the original dataset, as collected in electrode space. Each of the aforementioned tests was performed with each band-passed version of the EEG signals (*α*, *β*, *γ*) independently.

### Classification on source space

**Fig 6:**
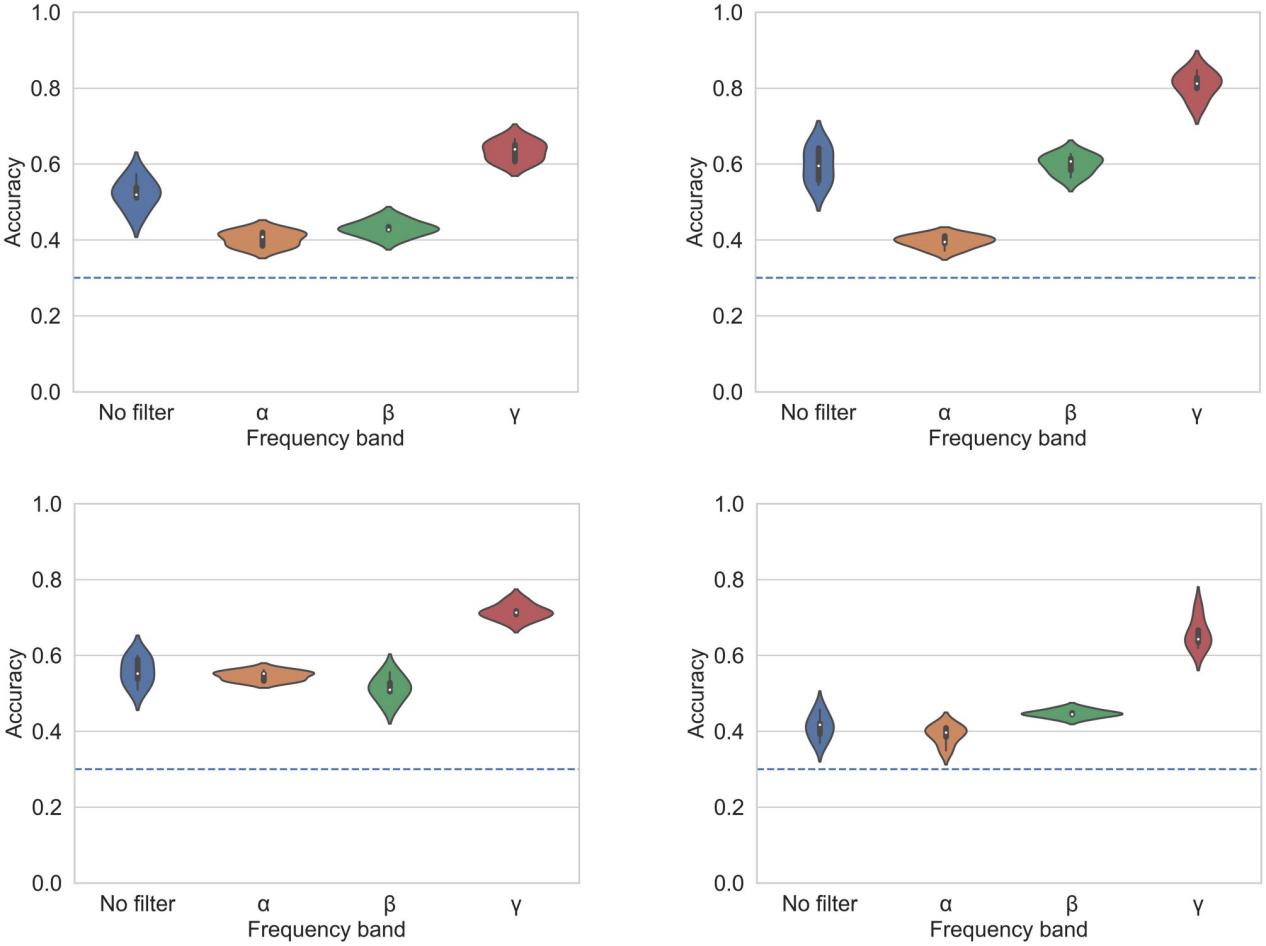
Classifier accuracies on source space. Accuracies of the topological classifier by frequency band on the space of sources without dimensionality reduction for participants 1, 3 (top right), 7 and 11. Results for all participants are shown in S1 Fig.

We performed the classification for the dataset of each participant within each frequency band, using the data projected onto source space prior to any dimensionality reduction. Fig 6 shows classification accuracies obtained by the TDA classifier for four typical participants—corresponding results for all participants are shown in S1 Fig.

Although the classification yielded some differences across participants, the main result obtained is a consistent top accuracy in the *γ* band for all participants but one, ranging in average between 60% to 80%. Violin plots in Fig 6 encode median, interquartile range, and a kernel-smoothed probability density of the data. The violin plots were performed using the seaborn library [55].

Using this as a baseline, we assessed the influence of dimensionality reduction on classification accuracy. For this, we applied two complementary dimensionality reduction techniques. For each participant dataset, we first performed a principal component analysis (PCA) decomposition, selecting the dimensions that would explain until 95% of data variance for all participants, ultimately retaining from 2 to 10 dimensions.

**Fig 7:**
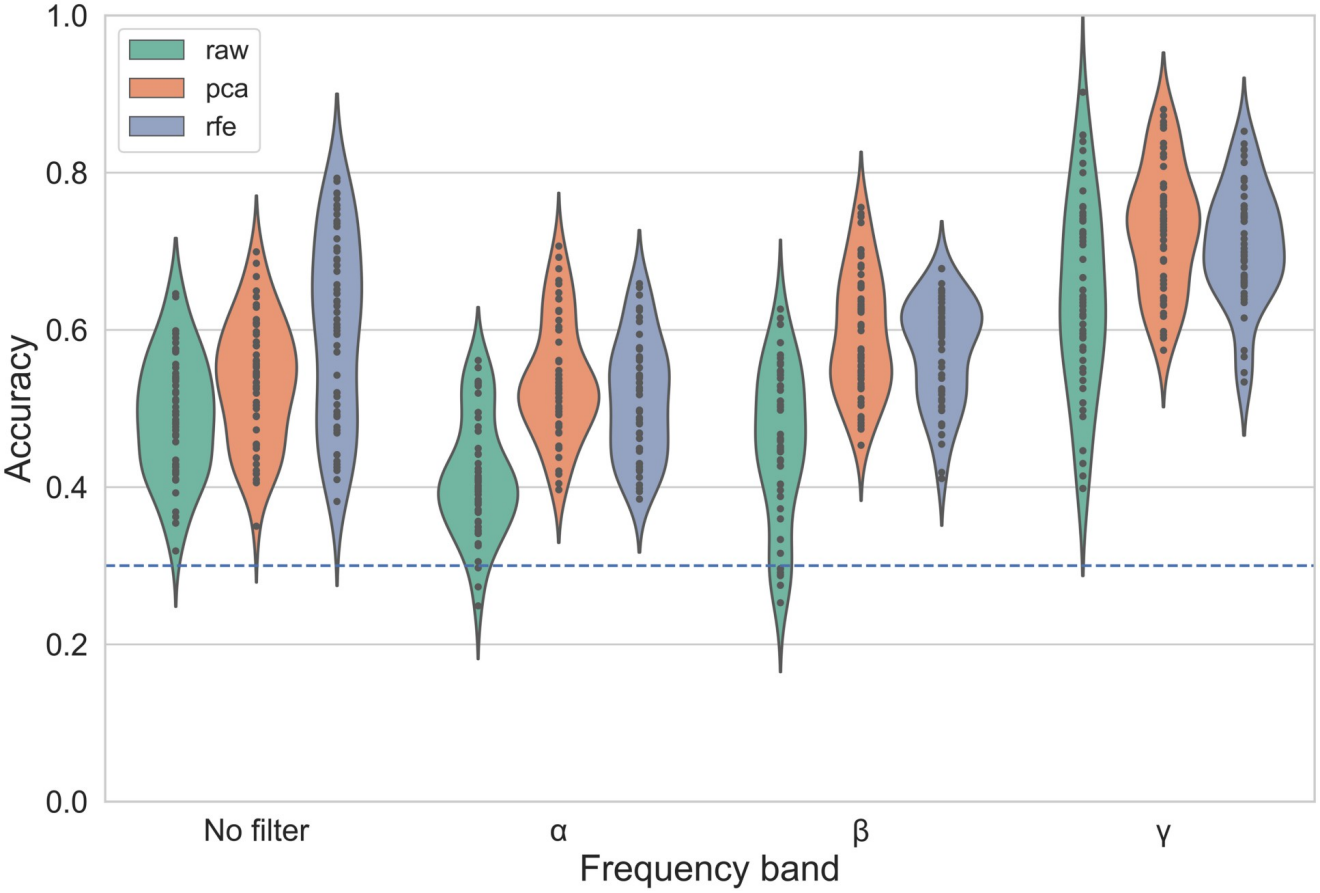
Violin plots of accuracies by frequency band. Comparison of baseline accuracies (*raw*) of the topological classifier on source space for each frequency band with accuracies obtained after dimensionality reduction with principal component analysis (*pca*) and recursive feature elimination (*rfe*), for participants 1 to 11. Each violin contains accuracy percentages of five repetitions for each participant.

In a complementary fashion, we performed recursive feature elimination (RFE) on the same datasets. Unlike PCA, recursive feature elimination is based on assessing the contribution of specific components of the original dataset to the classification process. When performed in source space, this results in a ranking of sources. The datasets resulting from PCA and RFE were also classified for each participant and for each frequency band. The summary results obtained from these classifications are shown as violin plots in Fig 7. Accuracy percentages are given in S6 Fig for each of the eleven participants, for each frequency band and for each dimensionality reduction method, after averaging the accuracies obtained from five repetitions in each dimension. Standard deviations are specified in S1 and S2 Tables. Since there are three distinct classes, chance level equals 0.33.

These analyses yielded two main results. First, consistently with the baseline classification results of the TDA classifier and with the results of previous machine learning classifiers, the highest accuracies were obtained in the *γ* band for all participants but one [36]. Second, consistently with the hypothesis of dimensionality reduction rearranging the point cloud in shapes that facilitate classification, our results consistently confirm that point clouds in reduced dimensionality spaces yield higher accuracies than the baseline, high-dimensional dataset.

### Effect of dimensionality reduction

Since higher classification accuracies are obtained in low dimensional spaces, the immediate question is how accuracy depends on dimensionality. In the case of RFE, we aimed at finding an optimal number of sources as guided by previous results from [36]. To this end, we tested our topological classifier using the space resulting of applying recursive feature elimination to the EEG data projected onto the space of sources, increasing the number of selected sources by the algorithm from 2 to 10. Classifications were performed for each participant and each frequency band.

Our thesis is that the notion of shape upon which TDA is based is essentially independent of data variability. Thus, we expected to find an optimal number of principal components such that the resulting projected spaces would yield shapes more favourable to be classified. We tested our topological classifier using the data clouds resulting from PCA applied to the space of sources, gradually increasing the number of selected principal components from 2 to 10. We recorded the classification accuracy as well as the amount of explained variance by the number of components at that step. Classifications were performed for each participant and for each frequency band in parallel. To further assess the effect of ICA to the shape of the data, we performed the same tests on the space of electrodes, namely the original EEG data prior to applying the ICA algorithm.

**Fig 8:**
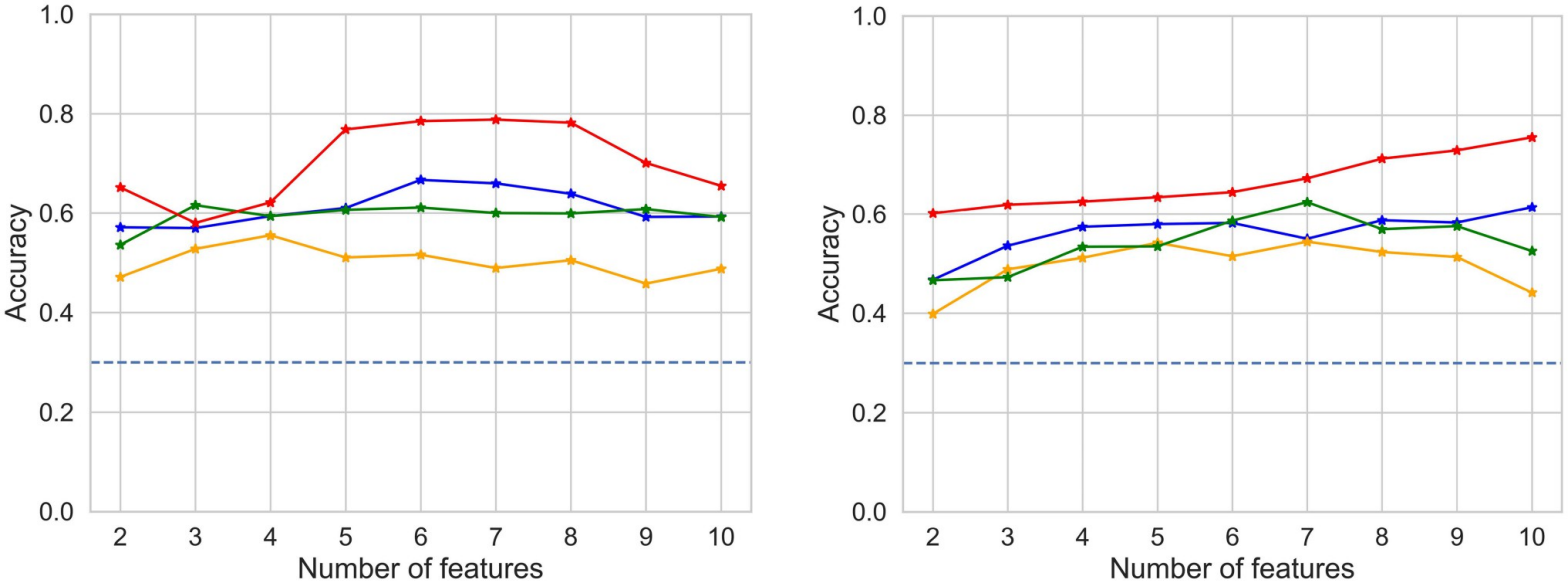
Classifier accuracy by frequency band. Variation of accuracy of the topological classifier by frequency band (blue: no filter; yellow: *α*; green: *β*; red: *γ*) depending on the number of sources picked by recursive feature selection for participants 1 and 8. The blue dotted line indicates chance level.

Fig 8 shows the accuracy of two typical participants as a function of the number of sources as selected by RFE (ranging from 2 to 10), for each frequency band. Results for all participants can be seen in S5 Fig. The highest accuracy is consistently obtained within the *γ* band. Furthermore, the accuracy shows that only a few sources are typically required to obtain the best accuracy—five sources suffice for the classification task for most participants, in accordance with results shown in [36].

**Fig 9:**
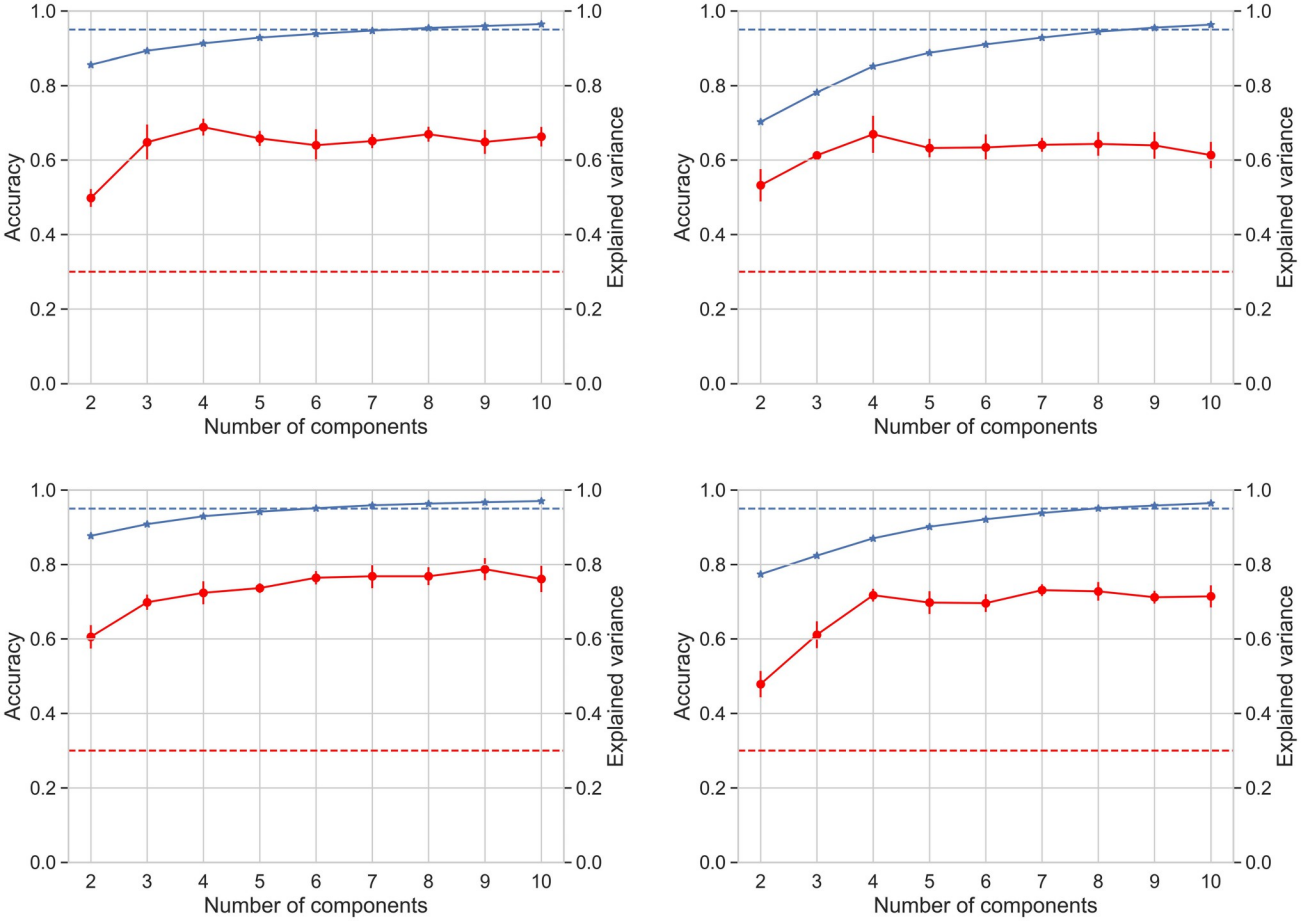
Classifier accuracy in a range of dimensions. Comparison of accuracy variation (red) with PCA explained variance (blue) as dimension increases for participants 1 (left) and 8 (right). The upper row corresponds to the space of electrodes and the lower row to the space of sources. The blue dotted line indicates 95% of explained variance and the red dotted line is chance level. Standard deviations of accuracy (red) are computed after five repetitions of the classifier. Graphs for all participants are shown in S3 and S4 Figs.

The top row of Fig 9 shows accuracy as a function of the number of dimensions considered when applying PCA to the original dataset (in electrode space). A peak is observed when the number of dimensions ranges around four. The bottom row in Fig 9 shows accuracy as a function of the number of dimensions when applying PCA to the dataset in source space. A full set of results for all participants is shown in S3 and S4 Figs. These results suggest that the increase of accuracy is essentially independent from the explained variance that accumulates when the number of PCA dimensions increases. This is clearer for the dataset corresponding to electrode space.

## Discussion

This study introduced a novel data classification algorithm based on principles derived from topological data analysis. By contrast to machine learning classifiers, typically based on assessing relative distance metrics across points of different classes, the topological classifier here described is based on exploiting differences in shape between point clouds from each class. Such differences were quantified by calculating dissimilarities between persistence silhouettes [51].

The stability theorem for persistence barcodes [40, 51] guarantees that small variations of a point cloud yield small variations on its persistence descriptors. Because of this, if a point cloud is structured into distinct classes—three in the case of our dataset—, the addition of a point to the class where it belongs should barely yield any effect on the resulting topological descriptors, while incorporating the same point to the point cloud of the other classes is likely to cause perceivable shape changes. This is the principle we here proposed to use for classification.

We tested this principle with an electro-encephalographic dataset recorded from human participants during a study of motivated behaviour [36], aimed at quantifying the influence of three levels of social pressure on the brain state of the human motivational system. As for most EEG analyses, the data time series were decomposed into three typical frequency bands: *α*, *β* and *γ* (see Methods), which we analysed alongside to an unfiltered (full frequency band) version of the dataset.

### Choice of dataset

The choice of dataset from [36] responded to several requirements:

1. The need of a rich, high-dimensional dataset, which contained data clouds of significant size. Our dataset had data from eleven participants, having performed 1296 trials each, distributed into three classes.
2. As a means to double validate our tests, it was convenient that the dataset had been previously analysed by reliable methods, as to offer a clear target of potential accuracies for the TDA classifier.
3. Previous analyses of this dataset have yielded classification accuracies increasing alongside with the frequency band considered, ranging from around 50–60% in the low *α* band to 75–90% in the *γ* band. This offered a range of results and an overall separability across datasets, which we could fall back on to assess the performance of the TDA classifier.

### Dimensionality of data

Our study was first performed with the space of electrodes and subsequently with a space of brain sources obtained from electrodes by means of independent component analysis (ICA). This technique is convenient from a statistical perspective because the data components in source space are mostly independent from each other. Furthermore, from the perspective of analysing neural data, it allows to estimate the brain source localization responsible for the signals recorded.

We tested the effect of dimensionality by assessing the performance of our TDA classifier within a range of dimensions by means of a principal component analysis (PCA) decomposition optimizing the explained variance of the data cloud. To ensure that we covered all cases until 95% variance, our PCA analysis gradually considered from two to ten dimensions. This yielded a remarkable result: while the degree of explained variance increased the accuracy asymptotically towards 100% even when considering over ten dimensions, the TDA classifier’s accuracy reached a plateau after a given boundary dimension—typically dimension four in the electrode space. This strongly suggests that the classifier’s operation is more sensitive to the latent dimension of the data cloud than to the amount of explained variance. This was reinforced by a comparative analysis with the performance of a nearest neighbour classifier, which monotonically increased its accuracy along with explained variance up to dimension ten (data not shown).

As an additional validation analysis of our dimensionality tests, we performed a recursive feature elimination decomposition (RFE) to identify the dimensions in source space contributing the most to the classification. This analysis yielded a similar ceiling effect on accuracy than the analysis based on dimensions ranked by explained variance of PCA, close to dimension five.

Our initial prediction was that a projection of the original dataset onto an independent component space (or source space) would yield a finer defined cloud and ultimately higher accuracies than those of the original dataset. This hypothesis is supported by our analysis about the influence of dimensionality with the ICA-dataset on the accuracy, which yielded lesser sensitivity when ICA was previously applied to the dataset. Likewise, our validation test with RFE also showed that there is a minimal number of necessary sources to obtain an asymptotic accuracy (typically five). The number of dimensions that effectively contributed to the classification with the TDA classifier matched the number of sources required to represent the brain network of motivation in the original study [36]. Moreover, the frequency band yielding the best results was the *γ* band, consistently with the referenced analysis.

Thus, our results suggest that the effect of the ambient dimension on persistence descriptors of a point cloud is of a different nature than its effect on data variance. As shown by Fig 9, S3 and S4 Figs, a higher ambient dimension entails greater variance of data and yet it is not necessarily associated with an improved accuracy of our topology-based classifier.

## Supporting information

S1 Fig Classifier accuracies. Accuracies of the TDA-based classifier by frequency band on the space of sources for participants 1 to 11 without dimensionality reduction.
(TIF)

S2 Fig Point clouds of brain states. Point clouds corresponding to participant 1 (left) and participant 8 (right), using the space of sources and the *γ* band, after applying PCA to obtain three-dimensional data clouds: *M*_0_ blue, *M*_1_ red, *M*_2_ green. The two clusters in each cloud correspond to the two sessions performed within each block.
(TIF)

S3 Fig PCA on sources. Comparison of variation of accuracy (red) with PCA explained variance (blue) as dimension increases for all participants on the space of sources within the *γ* frequency band. The blue dotted line indicates 95% of explained variance and the red dotted line is chance level. Standard deviations of accuracy (red) are computed after five repetitions of the classifier.
(TIF)

S4 Fig PCA on electrodes. Comparison of variation of accuracy (red) with PCA explained variance (blue) as dimension increases for all participants on the space of electrodes within the *γ* frequency band. The blue dotted line indicates 95% of explained variance and the red dotted line is chance level. Standard deviations of accuracy (red) are computed after five repetitions of the classifier.
(TIF)

S5 Fig RFE on sources. Comparison of variation of accuracy for each frequency band (blue: no filter; yellow: *α*; green: *β*; red: *γ*) as the number of sources increases from 2 to 10 using the RFE algorithm, for all participants on the space of sources. The blue dotted line is chance level.
(TIF)

S6 Fig Accuracy comparison. Comparison of baseline accuracies (*raw*) of the topological classifier on source space for each frequency band with accuracies obtained after dimensionality reduction with principal component analysis (*pca*) and recursive feature elimination (*rfe*), for participants 1 to 11. Highest accuracies are boldfaced.
(TIF)

S1 Table Classifier accuracies for *α* band. Comparison of baseline accuracies (*raw*) of the topological classifier on source space for each frequency filter with accuracies obtained after dimensionality reduction with principal component analysis (*pca*) and recursive feature elimination (*rfe*), with no frequency filter and in the *α* frequency band, for participants 1 to 11 with standard deviations after five repetitions.
(PDF)

S2 Table Classifier accuracies for *β* and *γ* bands. Comparison of baseline accuracies (*raw*) of the topological classifier on source space for each frequency filter with accuracies obtained after dimensionality reduction with principal component analysis (*pca*) and recursive feature elimination (*rfe*), in the *β* and *γ* frequency bands, for participants 1 to 11 with standard deviations after five repetitions.
(PDF)
