# Supplementary figures and images for: A topological classifier to characterize brain states: When shape matters more than variance
Source: PLoS One. 2023 Oct 2;18(10):e0292049. doi: 10.1371/journal.pone.0292049 (PMC10545107; doi:10.1371/journal.pone.0292049)

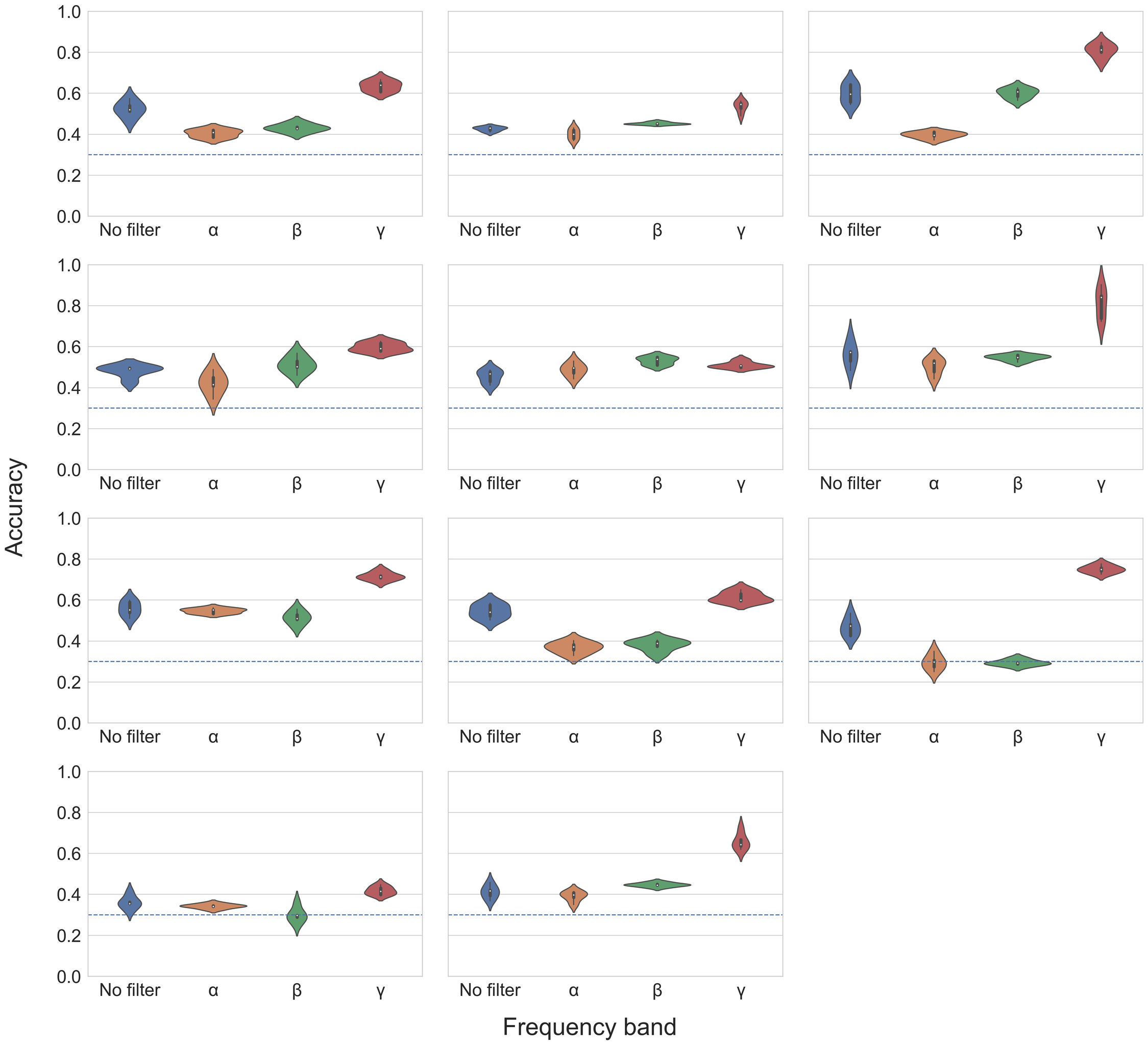

Supplement: S1 Fig — Accuracies of the TDA-based classifier by frequency band on the space of sources for participants 1 to 11 without dimensionality reduction. (TIF) [file pone.0292049.s001.tif]

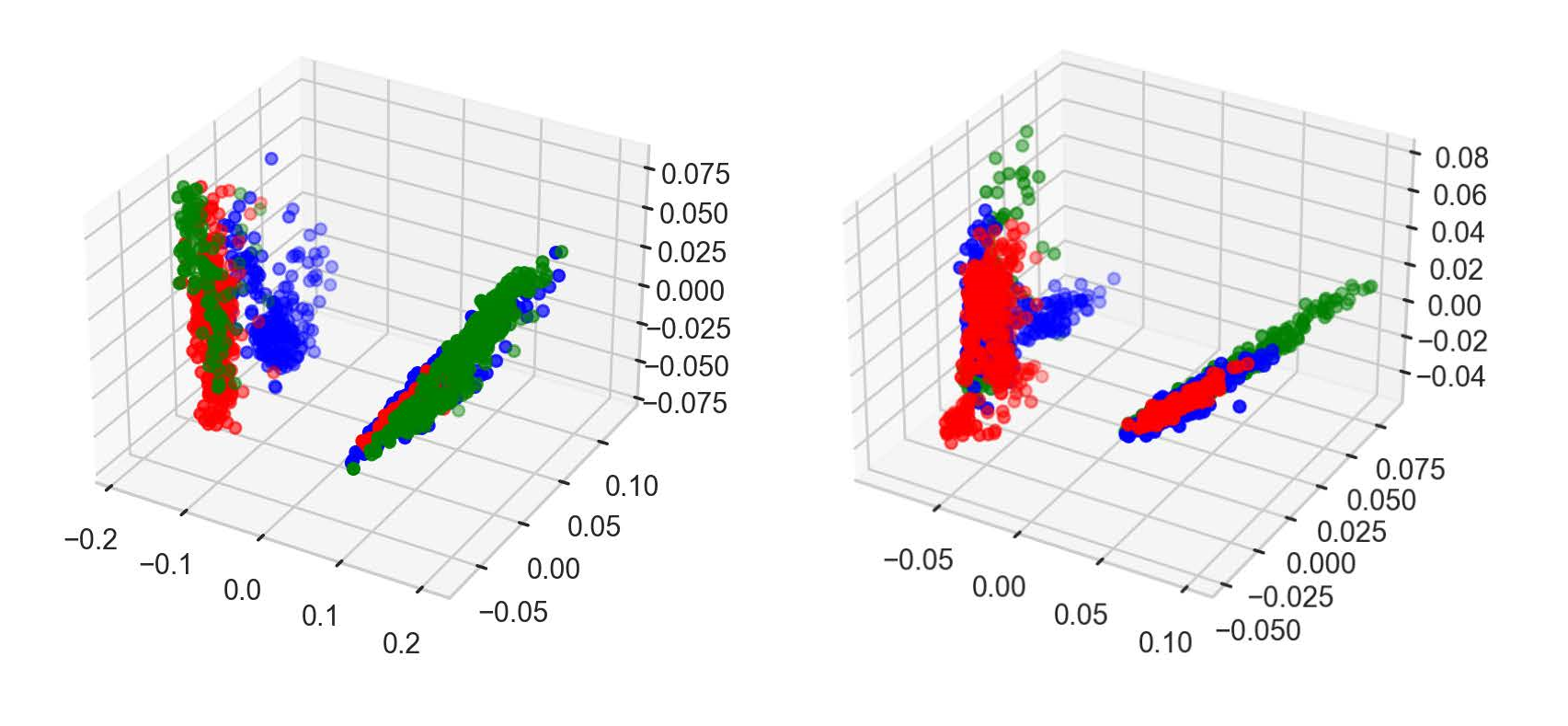

Supplement: S2 Fig — Point clouds corresponding to participant 1 (left) and participant 8 (right), using the space of sources and the γ band, after applying PCA to obtain three-dimensional data clouds: M0 blue, M1 red, M2 green. The two clusters in each cloud correspond to the two sessions performed within each block. (TIF) [file pone.0292049.s002.tif]

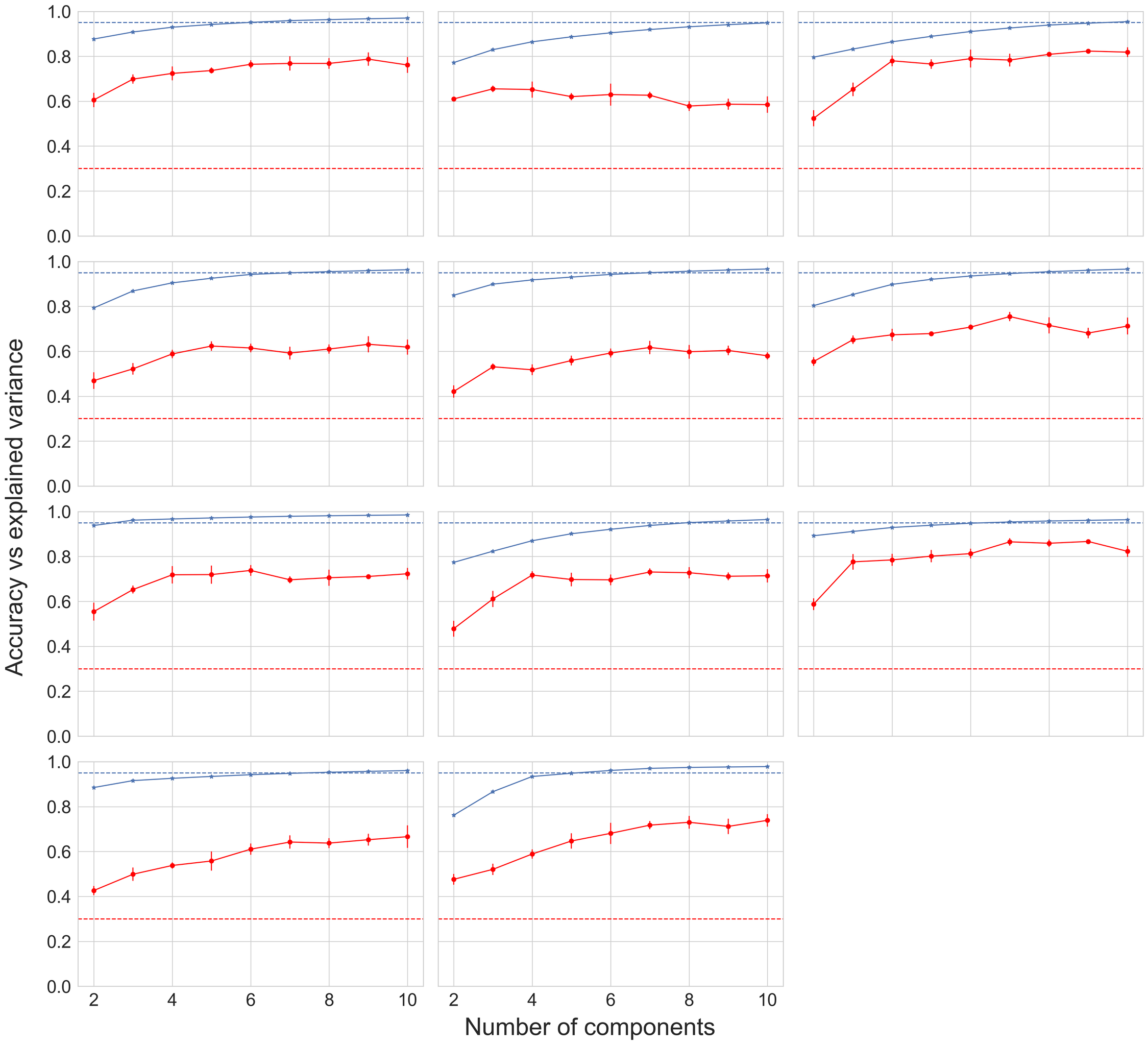

Supplement: S3 Fig — Comparison of variation of accuracy (red) with PCA explained variance (blue) as dimension increases for all participants on the space of sources within the γ frequency band. The blue dotted line indicates 95% of explained variance and the red dotted line is chance level. Standard deviations of accuracy (red) are computed after five repetitions of the classifier. (TIF) [file pone.0292049.s003.tif]

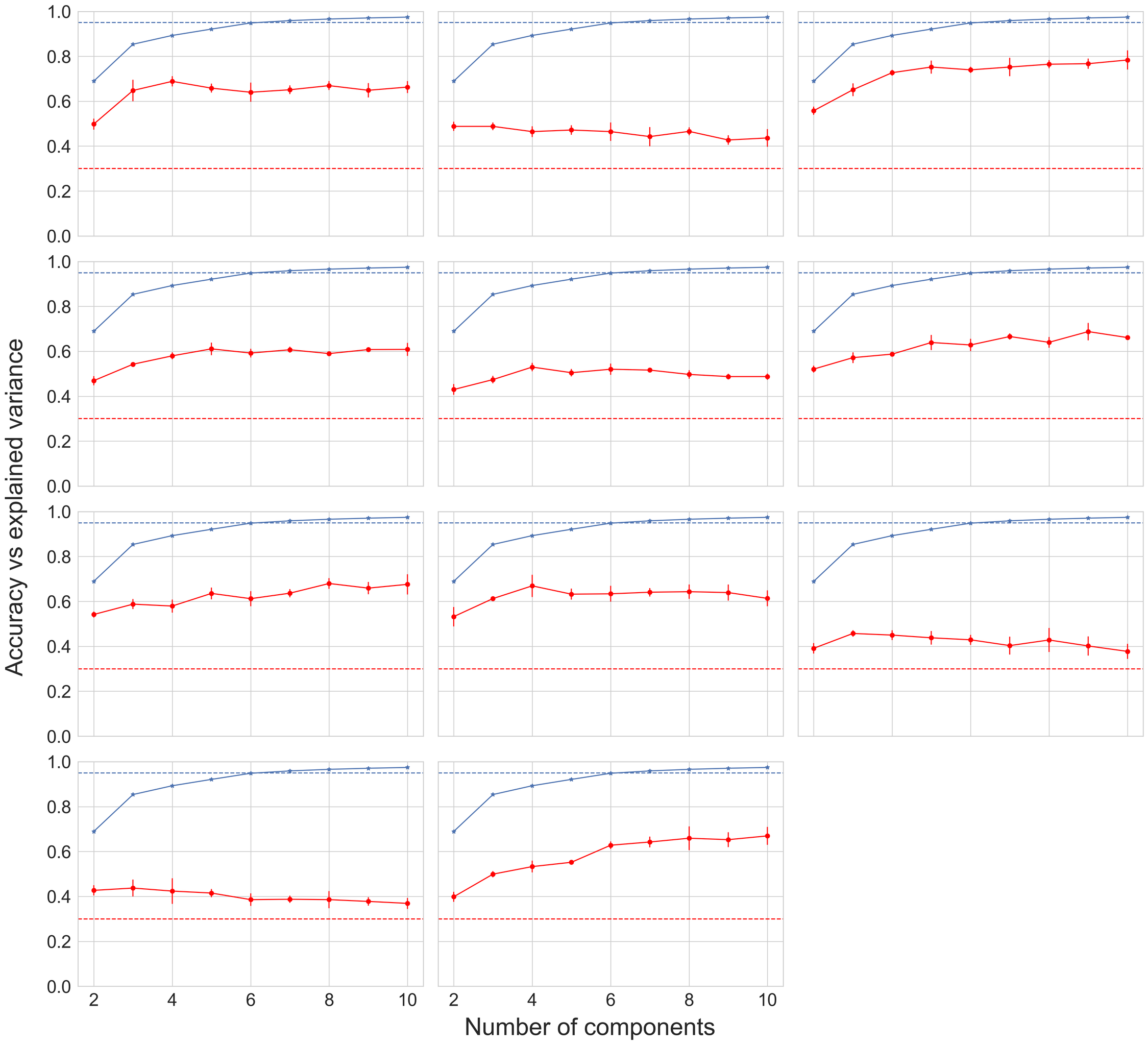

Supplement: S4 Fig — Comparison of variation of accuracy (red) with PCA explained variance (blue) as dimension increases for all participants on the space of electrodes within the γ frequency band. The blue dotted line indicates 95% of explained variance and the red dotted line is chance level. Standard deviations of accuracy (red) are computed after five repetitions of the classifier. (TIF) [file pone.0292049.s004.tif]

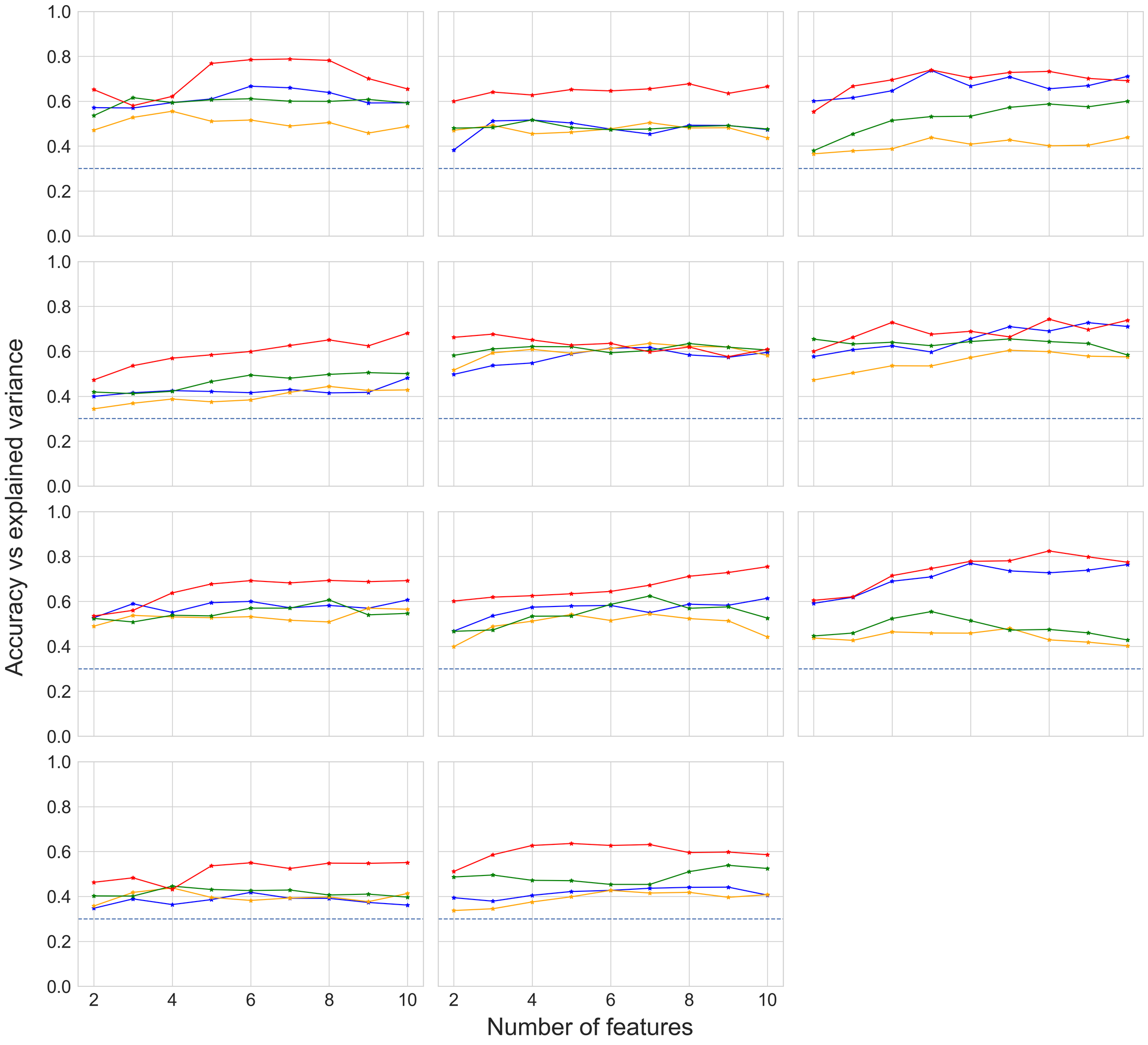

Supplement: S5 Fig — Comparison of variation of accuracy for each frequency band (blue: no filter; yellow: α; green: β; red: γ) as the number of sources increases from 2 to 10 using the RFE algorithm, for all participants on the space of sources. The blue dotted line is chance level. (TIF) [file pone.0292049.s005.tif]

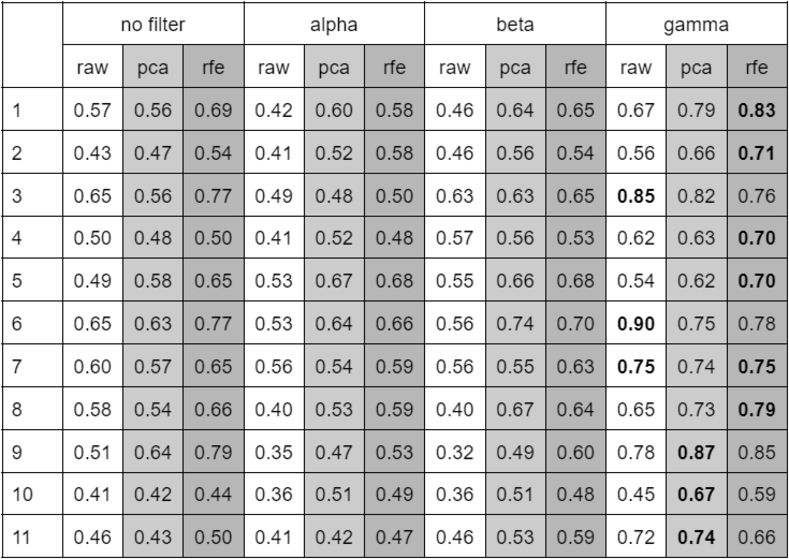

Supplement: S6 Fig — Comparison of baseline accuracies (raw) of the topological classifier on source space for each frequency band with accuracies obtained after dimensionality reduction with principal component analysis (pca) and recursive feature elimination (rfe), for participants 1 to 11. Highest accuracies are boldfaced. (TIF) [file pone.0292049.s006.tif]
