# Supplementary material for: A topological classifier to characterize brain states: When shape matters more than variance
Source: PLoS One. 2023 Oct 2;18(10):e0292049. doi: 10.1371/journal.pone.0292049 (PMC10545107; doi:10.1371/journal.pone.0292049)
Supplement: S2 Table — Comparison of baseline accuracies (raw) of the topological classifier on source space for each frequency filter with accuracies obtained after dimensionality reduction with principal component analysis (pca) and recursive feature elimination (rfe), in the β and γ frequency bands, for participants 1 to 11 with standard deviations after five repetitions. (PDF) [file pone.0292049.s008.pdf]

beta			gamma		
raw	pca	rfe	raw	pca	rfe
0.46 ± 0.02	0.64 ± 0.03	0.65 ± 0.03	0.67 ± 0.03	0.79 ± 0.03	0.83 ± 0.08
0.46 ± 0.01	0.56 ± 0.03	0.54 ± 0.03	0.56 ± 0.03	0.66 ± 0.01	0.71 ± 0.04
0.63 ± 0.03	0.63 ± 0.02	0.65 ± 0.07	0.85 ± 0.03	0.82 ± 0.01	0.76 ± 0.06
0.57 ± 0.04	0.56 ± 0.02	0.53 ± 0.04	0.62 ± 0.02	0.63 ± 0.04	0.70 ± 0.07
0.55 ± 0.02	0.66 ± 0.04	0.68 ± 0.02	0.54 ± 0.02	0.62 ± 0.03	0.70 ± 0.04
0.56 ± 0.01	0.74 ± 0.02	0.70 ± 0.03	0.90 ± 0.08	0.75 ± 0.02	0.78 ± 0.05
0.56 ± 0.03	0.55 ± 0.01	0.63 ± 0.04	0.75 ± 0.02	0.74 ± 0.02	0.75 ± 0.06
0.40 ± 0.03	0.67 ± 0.02	0.64 ± 0.05	0.65 ± 0.03	0.73 ± 0.02	0.79 ± 0.06
0.32 ± 0.01	0.49 ± 0.03	0.60 ± 0.05	0.78 ± 0.02	0.87 ± 0.01	0.85 ± 0.08
0.36 ± 0.04	0.51 ± 0.03	0.48 ± 0.03	0.45 ± 0.02	0.67 ± 0.05	0.59 ± 0.05
0.46 ± 0.01	0.53 ± 0.02	0.59 ± 0.04	0.72 ± 0.04	0.74 ± 0.03	0.66 ± 0.04
