# Supplementary material for: A topological classifier to characterize brain states: When shape matters more than variance
Source: PLoS One. 2023 Oct 2;18(10):e0292049. doi: 10.1371/journal.pone.0292049 (PMC10545107; doi:10.1371/journal.pone.0292049)
Supplement: Attachment — Submitted filename: Response_to_Reviewers.pdf [file pone.0292049.s009.pdf]

UNIVERSITAT DE BARCELONA

Faculty of Mathematics and Computer Science
Gran Via de les Corts Catalanes, 585
08007 Barcelona
Tel. +34 93 402 1605
carles.casacuberta@ub.edu

Rebuttal letter for PONE-D-23-06017

Barcelona, May 20, 2023

Dear Editor,

Please find attached our responses to the indications of the reviewers of our manuscript entitled *A topological classifier to characterize brain states: When shape matters more than variance*.

The manuscript has been fully revised accordingly. We appreciate very much the suggestions made by the reviewers and have taken all of them into account. As a result, the structure of the article has changed and large parts of it have been rewritten. Two figures have been added and the overall quality of the figures has been improved. Although it is difficult to specify in the manuscript all the changes in detail, we have marked in blue color the parts that have been substantially modified.

We have also followed the indications of the Academic Editor. In order to facilitate reproducibility of our study, the datset has been uploaded to Kaggle:

https://www.kaggle.com/ds/3333171, with DOI 10.34740/kaggle/ds/3333171,

and code is now available at a public github repository:

github.com/ainafem/brain_states_motivation.

We very much hope that after this version the article fully meets PLOS ONE's publication criteria.

Sincerely yours,

Aina Ferrà
Gloria Cecchini
Fritz-Pere Nobbe Fisas
Carles Casacuberta
Ignasi Cos

Answers to Reviewer 1

1. **From the current manuscript, it is hard to understand the intuition why topology is beneficial. Furthermore, clarity of the manuscript can be improved.**

   The structure of the manuscript has been changed by switching the ordering of subsections in the Materials and Methods section, and many paragraphs have been rewritten. We hope that this revised version meets the criteria and standards of the journal.

   Topological features of data are the basis of the classifier proposed in this article. It is now more clearly explained in the Topological Classifier subsection that our underlying assumption is that point clouds sampled from different brain states exhibit recognizably different shapes. The plausibility of this claim has been tested by means of bootstrapping on each motivational state. The resulting distributions of zero-dimensional total persistence are significantly different, as now shown in Fig. 3 and ascertained with a Kolmogorov–Smirnov test with p-values below 0.0001. Further evidence of the difference between topological descriptors of the three motivational states is given by the silhouettes depicted in Fig. 4.

2. **After PCA, 2-10 dimensional vectors are given. Then, how do you compute the landscape? What corresponds to the "point clouds" in TDA?**

   Persistence landscapes are computed from persistence diagrams of Vietoris–Rips simplicial complexes calculated from point clouds equipped with Euclidean distance. Point clouds represent vectors extracted from the dataset. The dimension of each point cloud is the dimension of the ambient space, i.e., the number of channels —electrodes or brain sources—, and there is a point for each of 12 blocks of 108 trials per participant. After PCA reduction, the dimension of the ambient space decreases accordingly. Thus, Vietoris–Rips simplicial complexes are computed anew using vectors of the corresponding reduced dimension, and landscapes are then derived from the resulting persistence diagrams.

3. **Post processing is still ambiguous to me. The data length of the band spectra or time series is probably not explicitly given. (The width of time bins are not specified.) That dimension size should give a nice intuition if the classification works or not. I would like to see a figure for point clouds, for example.**

   The dimension size of the EEG dataset is defined by the number of electrodes placed on the headcap (60). Each electrode records brain activity generated across the brain, but perceivable through one small area on the participant's skull, where the electrode is placed. As described in the original neuroscience study (Cos et al. 2021), the signals were sampled at 1 KHz at the time of recording during an interval of 1200 ms. The choice of this interval responds to a combination of technical and scientific constraints. First, EEG activity is heavily corrupted by movement artifacts as soon as the movement response is initiated (and sometimes prepared) to the extent that its analysis becomes impossible. Second, the purpose of the original neuroscience study was to observe baseline modulations of social pressure on the participant's brain state, which could only be reliably assessed prior to each trial presentation of options. This is fully explained in Cos et al. 2021.

   That all being said, the dataset provided for the present article had been time averaged after independent component analysis and bandpass filter processing. Hence, there is no temporal dimension to consider, but only the number of electrodes (60), the number of trials (432), and the number of brain states (3).

   Two point clouds, corresponding to participants 1 and 8 in the study after PCA reduction to dimension 3, are now shown in Fig. 11. The shape distinction between the three motivational states M_0, M_1, M_2 can be clearly seen in this image.

4. **Each figure is dirty (Figure 3 and later). The labels are too small to read. No label is given on the x- and y- axes in Figure 3. (Units are not available.)**

   We have rebuilt all the images to achieve higher resolution. Furthermore, we have increased the font sizes of labels and units in the figures, and ensured that units are specified in all diagram axes where units are pertinent. The case of Fig. 3 is special since units in the axes of persistence silhouettes do not have any standard interpretation. To remedy this as much as possible, we have specified the meaning of axes in the caption of Fig. 3, as follows: The x-axis represents lifetime lengths of connected components of the Vietoris–Rips complex, normalized into 1000 vector units by equating ranges. The y-axis represents density of points in the persistence diagram weighted by their lifetime, uniformized by equating height in the pictures.

Answers to Reviewer 2

1. **The information to reproduce the results from the manuscript is lacking, and there are also leaps in the discussion. Therefore, it is considered necessary to substantially revise the manuscript about these points.**

   The dataset used in this study has been uploaded into Kaggle and our code has been uploaded to a GitHub repository. The URLs are indicated below and in page 15 of the manuscript.

   The Discussion has been rewritten with substantial changes, striving for more clarity and avoiding speculative claims not fully supported by numerical results in the article. Other parts of the draft have also been rewritten; further details are now given in the Materials and Methods section, and two new figures have been added (Fig. 3 and Fig. 11).

2. **[lines 28 - 37] Only [27] and [28] are mentioned as previous studies of classification methods using TDA, but there are likely other methods. For example, I found the following ones just by doing a short search: [Genki Kusano, Yasuaki Hiraoka, Kenji Fukumizu Proceedings of The 33rd International Conference on Machine Learning, PMLR 48:2004-2013, 2016.] A more elaborate survey of previous studies should be conducted and the differences between them and the present method should be clearly explained.**

   The article by Kusano–Hiraoka–Fukumizu about a persistence weighted Gaussian kernel for topological data analysis has been added to the References of the article and is now mentioned in line 27 of the Introduction, together with articles by Bubenik and Bubenik–Dłotko about persistence landscapes. These articles contain indeed important examples of topological descriptors that have proved to be powerful tools for statistical analyses. In the article by Kusano–Hiraoka–Fukumizu, persistence weighted Gaussian kernels are used to achieve an improved accuracy in a support vector machine classifier.

   The classifier described in our article does not require the use of support vector machines nor of any other machine learning classifier, but it relies on topological descriptors from data. As far as we know, ours is the first article in which a classifier based solely on topological data analysis has been used with experimental data. A precedent that has to be mentioned is the article by Kindelan–Frías–Cerda–Hitschfeld (2021), where Vietoris–Rips simplicial complexes computed from data clouds were used as a refinement of nearest neighbour classifiers, which is very distinct from our method. Our classifier is similar to the one used in Inés–Domínguez–Heras–Mata–Rubio (2023) for semi-supervised machine learning tasks. The purpose of their article is different from ours in that they do not use persistence silhouettes as topological descriptors, and the aim of their work is not related to ours.

3. **The reproducibility cannot be checked because the values of all hyperparameters of the methods used in the analysis nor the methods used to determine them are not described in the manuscript. For example, the criteria for noisy channels [line 248], the shape of the window function of the bandpass filter [line 258], and the**

hyperparameters for "kernel-smoothed" [line 307] should be described. Also, it is highly recommended that the code and data used in the manuscript should be made publicly available to confirm the reproducibility.

The filter used to band-pass the original temporal series was a fourth-order Butterworth filter with boundary frequencies $8 - 15$ Hz for the alpha band, $15 - 32$ Hz for the beta band, and $32 - 80$ Hz for the gamma band. Details have been added to our account of the signal processing (lines 114–122 and 277–278). Kernel-smoothing was not used in our analyses; it is only mentioned in order to ease interpretation of violins depicted in Fig. 6 and subsequently. Default parameters from the Seaborn library were used to draw violins.

That being said, we note that time series for this study were provided already averaged in the dataset. We have not performed any bandpass filtering nor any smoothing when carrying out analyses in the article under consideration. Although the focus of our study is the TDA classifier, we considered appropriate to provide an account of the data-conditioning process for completeness. The full specifics of the original data conditioning are available in a previous neuroscience study (Cos et al. 2021).

In order to ensure that data used in the manuscript be publicly available for reproducibility purposes, the dataset has been uploaded to Kaggle:

`https://www.kaggle.com/ds/3333171`,

and our code has been uploaded to a public GitHub repository:

`https://github.com/ainafem/brain_states_motivation`.

4. **The claims that the proposed method captures geometric structure rather than data variance in general data seem inappropriate as claims deduced from the results of numerical experiments. Therefore, numerical experiments or theoretical analysis supporting such claims, or the withdrawal of the claims, would be necessary for the publication of the manuscript.**

   It is true that the concept of "explained shape" as opposed to explained variance that we mentioned in the previous version of the article was not defined in a precise way and was speculative. Therefore we have removed every reference to explained shape from the manuscript. However, the concept of shape of a point cloud is defined rigorously in the framework of topological data analysis —namely, it refers to persistent homology of Vietoris–Rips complexes associated with a given finite set of points in Euclidean space. The classifier that we use in this article is based on comparison of silhouettes drawn from persistence diagrams of Vietoris–Rips complexes and is consequently strongly based on topological invariants of such complexes.

   In other words, the outcome of our classifier does not directly depend on the variance of the given datasets, but it is derived instead from topological descriptors of data. Furthermore, our results in the article (specifically, Figures 9, 12 and 13) demonstrate that the accuracy of our classifier remains stable in most cases along a range of dimensions for which variance changes steadily (as implicit in principal component analysis).

   We have rewritten the Results section and the Discussion in order to describe this fact more clearly. In addition, we have added Fig. 3, showing that shape descriptors computed from Vietoris–Rips simplicial complexes are significantly different in the three motivational states studied in the article. This justifies an a priori plausibility of the effectiveness of a classifier based on shape differences between such motivated brain states.

5. **[line 59 and 62] Why exclude the cases $d = 1$ and $k = 0$ even though 0-dimensional holes are analyzed as described in line 117?**

   Although the case $d = 1$ is usually omitted in topological data analysis of point clouds, it is true that all definitions make sense for $d = 1$ and we have therefore included the case $d = 1$ in our presentation of persistence descriptors (in line 156). As for $k = 0$, the 0-simplices are precisely the vertices of the Vietoris–Rips complex and we have made this more explicit in line 159.

6. **[line 63] It would be good to have an explanation as to why there are 2 in 2*t*.**

   The $2t$ value is usually chosen for the sake of consistency with the simplicial set $C_t(X)$ obtained from the *Čech filtration*, in which a k-simplex with vertices $v_0, \ldots, v_k$ occurs when closed balls of radius t centered at $v_0, \ldots, v_k$ have a common intersection (since this happens when the distance between their centres is lower than $2t$). However, Čech filtrations are not used in our article, and for our purposes it is indistinct to use t or $2t$. Therefore, we have now written $\|v_i - v_j\| \leq t$ for simplicity in line 160 and adjusted persistence values accordingly.

7. **[line 67] I think an explanation of the definition of F11 is needed. If it means a finite body of degree 2, is it not more common to use F2?**

   The notation $\mathbb{F}_{11}$ was meant to denote a finite field of 11 elements. While this choice of 11 may seem peculiar, it is the default choice used by the GUDHI software. This choice is plausible since $1 \neq -1$ for odd primes (which would be relevant if torsion phenomena were meaningful for analysis), and the prime number $p = 11$ is allegedly well suited in geometric or number-theoretic contexts.

8. **[line 149 and others] The definition of word "shape" is unclear. It would be easier for the reader to consider the effectiveness of the algorithm if it were made explicit about what structure is assumed.**

   This part of the Materials and Methods section has been rewritten, and Fig. 3 has been added to illustrate the meaning of shape distinctions between brain states within the data cloud. Indeed, "shape" is quantified by descriptors from persistence diagrams, such as total persistence, landscapes or silhouettes.

9. **[Fig. 4] An explanation of the abbreviation Mot. St. was not found anywhere in the manuscript and should be mentioned somewhere.**

   The abbreviation "Mot. St." appears in Fig. 5. The meaning of all abbreviations used in Fig. 5 has been specified in the caption.

10. **[Fig. 5] It should explain why it is classified on a per-subject basis.**

    The dataset used to test our TDA classifier was recorded in Cos et al. 2021. Their goal was to characterize the motivated brain state of each participant as a function of the manipulation of social pressure. Hence, the original analysis was made on a single-participant basis.

    The interest in making single-participant analysis is bound to two main reasons: First, it is due to the fact that each recording stems from a different brain, with significant differences in substrate. Second, with an eye on the possibility of using these techniques with clinical patients, it was of significant relevance to show that analyses were successful on single-participant data.

11. **[lines 309-316] The explanation of this paragraph was unclear to me so it would improve readability if it were rewritten for clarity.**

    This paragraph has been rewritten and it now reads as follows, hopefully more clearly: "Using this as a baseline, we assessed the influence of dimensionality reduction on classification accuracy. For this, we applied two complementary dimensionality reduction techniques. For each participant dataset, we first performed a principal component analysis (PCA) decomposition, selecting the dimensions that would explain until 95% of data variance for all participants, ultimately retaining from 2 to 10 dimensions."
