# Supplementary material for: A topological classifier to characterize brain states: When shape matters more than variance
Source: PLoS One. 2023 Oct 2;18(10):e0292049. doi: 10.1371/journal.pone.0292049 (PMC10545107; doi:10.1371/journal.pone.0292049)
Supplement: Attachment — Submitted filename: response_to_reviewers.pdf [file pone.0292049.s010.pdf]

UNIVERSITAT DE BARCELONA

Faculty of Mathematics and Computer Science
Gran Via de les Corts Catalanes, 585
08007 Barcelona
Tel. +34 93 402 1605
carles.casacuberta@ub.edu

Rebuttal letter for PONE-D-23-06017

Barcelona, June 23, 2023

Dear Editor,

At your request, please find attached a marked copy of our manuscript *A topological classifier to characterize brain states: When shape matters more than variance*, in which the changes made in the previous manuscript submitted on March 1 have been highlighted more clearly than in our previous revision dated May 20. In the current version, deleted text is crossed out and marked in red, while added text is marked in blue. In addition, a few comments are marked in brown.

As for Competing Interests, we state the following sentence according to your instructions:

The authors have declared that no competing interests exist.

Sincerely yours,

Aina Ferrà
Gloria Cecchini
Fritz-Pere Nobbe Fisas
Carles Casacuberta
Ignasi Cos
