# Supplementary material for: A topological classifier to characterize brain states: When shape matters more than variance
Source: PLoS One. 2023 Oct 2;18(10):e0292049. doi: 10.1371/journal.pone.0292049 (PMC10545107; doi:10.1371/journal.pone.0292049)
Supplement: Peer review history

# Peer review: Decision Letter 0

**DOI:** 10.1371/journal.pone.0292049.r001

**Authors:** Ruriko Yoshida

5 Apr 2023

PONE-D-23-06017A topological classifier to characterize brain states: When shape matters more than variancePLOS ONE

Dear Dr. Ferrà,

Thank you for submitting your manuscript to PLOS ONE. After careful consideration, we feel that it has merit but does not fully meet PLOS ONE’s publication criteria as it currently stands. Therefore, we invite you to submit a revised version of the manuscript that addresses the points raised during the review process.

One referee commented that you need add more information on reproducibility.  Please see their comments attached for more details.

Please submit your revised manuscript by May 20 2023 11:59PM. If you will need more time than this to complete your revisions, please reply to this message or contact the journal office at plosone@plos.org. When you're ready to submit your revision, log on to https://www.editorialmanager.com/pone/ and select the 'Submissions Needing Revision' folder to locate your manuscript file.

Please include the following items when submitting your revised manuscript:

- A rebuttal letter that responds to each point raised by the academic editor and reviewer(s). You should upload this letter as a separate file labeled 'Response to Reviewers'.
- A marked-up copy of your manuscript that highlights changes made to the original version. You should upload this as a separate file labeled 'Revised Manuscript with Track Changes'.
- An unmarked version of your revised paper without tracked changes. You should upload this as a separate file labeled 'Manuscript'.

If you would like to make changes to your financial disclosure, please include your updated statement in your cover letter. Guidelines for resubmitting your figure files are available below the reviewer comments at the end of this letter.

If applicable, we recommend that you deposit your laboratory protocols in protocols.io to enhance the reproducibility of your results. Protocols.io assigns your protocol its own identifier (DOI) so that it can be cited independently in the future. For instructions see: https://journals.plos.org/plosone/s/submission-guidelines#loc-laboratory-protocols. Additionally, PLOS ONE offers an option for publishing peer-reviewed Lab Protocol articles, which describe protocols hosted on protocols.io. Read more information on sharing protocols at https://plos.org/protocols?utm_medium=editorial-email&utm_source=authorletters&utm_campaign=protocols.

We look forward to receiving your revised manuscript.

Kind regards,

Ruriko Yoshida

Academic Editor

PLOS ONE

Journal Requirements:

When submitting your revision, we need you to address these additional requirements.

1. Please ensure that your manuscript meets PLOS ONE's style requirements, including those for file naming. The PLOS ONE style templates can be found at https://journals.plos.org/plosone/s/file?id=wjVg/PLOSOne_formatting_sample_main_body.pdf and 

https://journals.plos.org/plosone/s/file?id=ba62/PLOSOne_formatting_sample_title_authors_affiliations.pdf

2. Thank you for stating the following in your Competing Interests section:  

"None"

Please complete your Competing Interests on the online submission form to state any Competing Interests. If you have no competing interests, please state "The authors have declared that no competing interests exist.", as detailed online in our guide for authors at http://journals.plos.org/plosone/s/submit-now 

 This information should be included in your cover letter; we will change the online submission form on your behalf.

3. We note that you have stated that you will provide repository information for your data at acceptance. Should your manuscript be accepted for publication, we will hold it until you provide the relevant accession numbers or DOIs necessary to access your data. If you wish to make changes to your Data Availability statement, please describe these changes in your cover letter and we will update your Data Availability statement to reflect the information you provide.

One referee commented that you need to add more information for reproducibility. Please see referees' comments for more details.

[Note: HTML markup is below. Please do not edit.]

Reviewers' comments:

Reviewer's Responses to Questions

**Comments to the Author**

1. Is the manuscript technically sound, and do the data support the conclusions?

The manuscript must describe a technically sound piece of scientific research with data that supports the conclusions. Experiments must have been conducted rigorously, with appropriate controls, replication, and sample sizes. The conclusions must be drawn appropriately based on the data presented. 

Reviewer #1: Yes

Reviewer #2: Partly

**********

2. Has the statistical analysis been performed appropriately and rigorously? 

Reviewer #1: Yes

Reviewer #2: Yes

**********

3. Have the authors made all data underlying the findings in their manuscript fully available?

The PLOS Data policy requires authors to make all data underlying the findings described in their manuscript fully available without restriction, with rare exception (please refer to the Data Availability Statement in the manuscript PDF file). The data should be provided as part of the manuscript or its supporting information, or deposited to a public repository. For example, in addition to summary statistics, the data points behind means, medians and variance measures should be available. If there are restrictions on publicly sharing data—e.g. participant privacy or use of data from a third party—those must be specified.

Reviewer #1: Yes

Reviewer #2: No

**********

4. Is the manuscript presented in an intelligible fashion and written in standard English?

PLOS ONE does not copyedit accepted manuscripts, so the language in submitted articles must be clear, correct, and unambiguous. Any typographical or grammatical errors should be corrected at revision, so please note any specific errors here.

Reviewer #1: Yes

Reviewer #2: No

**********

5. Review Comments to the Author

Please use the space provided to explain your answers to the questions above. You may also include additional comments for the author, including concerns about dual publication, research ethics, or publication ethics. (Please upload your review as an attachment if it exceeds 20,000 characters)

Reviewer #1: The authors used topology for classifying the EEG states.

The TDA method using landscapes looks sound and novel.

I found the result is new enough.

However, from the current manuscript, it is hard to understand the intuition why topology is beneficial.

Furthermore, clarity of the manuscript can be improved.

(1) After PCA, 2-10 dimensional vectors are given. Then, how do you compute the landscape? What corresponds to the “point clouds” in TDA.

(2) Post processing is still ambiguous to me.

The data length of the band spectra or time series is probably not explicitly given. (The width of time bins are not specified.)

That dimension size should give a nice intuition if the classification works or not.

I would like to see a figure for point clouds, for example.

(3) Each figure is dirty (Figure 3 and later).

The labels are too small to read.

No label is given on the x- and y- axes in Figure 3. (Units are not available.)

Reviewer #2: The manuscript proposes an algorithm for data classification by combining clustering and topological data analysis. The use of topological features, rather than distances from clustering centers, for classifying test data using clustering results is novel. However, The information to reproduce the results from the manuscript is lacking, and there are also leaps in the discussion. Therefore, it is considered necessary to substantially revise the manuscript about these points.

The detailed major concerns are described as follows.

1. [lines 28 - 37] Only [27] and [28] are mentioned as previous studies of classification methods using TDA, but there are likely other methods. For example, I found the following ones just by doing a short search.

[Genki Kusano, Yasuaki Hiraoka, Kenji Fukumizu Proceedings of The 33rd International Conference on Machine Learning, PMLR 48:2004-2013, 2016.]

A more elaborate survey of previous studies should be conducted and the differences between them and the present method should be clearly explained.

2. The reproducibility cannot be checked because the values of all hyperparameters of the methods used in the analysis nor the methods used to determine them are not described in the manuscript.

For example, the criteria for noisy channels [line 248], the shape of the window function of the bandpass filter [line 258], and the hyperparameters for "kernel-smoothed" [line 307] should be described.

Also, it is highly recommended that the code and data used in the manuscript should be made publicly available to confirm that the reproducibility

3. The claims that the proposed method captures geometric structure rather than data variance in general data seem inappropriate as claims deduced from the results of numerical experiments. Therefore, numerical experiments or theoretical analysis supporting such claims, or the withdrawal of the claims, would be necessary for the publication of the manuscript.

The minor concerns are described below.

1. [line 59 and 62] Why exclude the cases d=1 and k=0 even though 0-dimensional holes are analyzed as described in line 117?

2. [line 63] It would be good to have an explanation as to why there are 2 in 2t.

3. [line 67] I think an explanation of the definition of F11 is needed. If it means a finite body of degree 2, is it not more common to use F_2?

4. [line 149 and others] The definition of word "shape" is unclear. It would be easier for the reader to consider the effectiveness of the algorithm if it were made explicit about what structure is assumed.

5. [Fig. 4] An explanation of the abbreviation Mot. St. was not found anywhere in the manuscript and should be mentioned somewhere.

6. [Fig. 5] It should explain why it is classified on a per-subject basis.

7. [lines 309-316] The explanation of this paragraph was unclear to me so it would improve readability if it were rewritten for clarity.

**********

6. PLOS authors have the option to publish the peer review history of their article (what does this mean?). If published, this will include your full peer review and any attached files.

If you choose “no”, your identity will remain anonymous but your review may still be made public.

**Do you want your identity to be public for this peer review?** For information about this choice, including consent withdrawal, please see our Privacy Policy.

Reviewer #1: No

Reviewer #2: No

**********

[NOTE: If reviewer comments were submitted as an attachment file, they will be attached to this email and accessible via the submission site. Please log into your account, locate the manuscript record, and check for the action link "View Attachments". If this link does not appear, there are no attachment files.]

While revising your submission, please upload your figure files to the Preflight Analysis and Conversion Engine (PACE) digital diagnostic tool, https://pacev2.apexcovantage.com/. PACE helps ensure that figures meet PLOS requirements. To use PACE, you must first register as a user. Registration is free. Then, login and navigate to the UPLOAD tab, where you will find detailed instructions on how to use the tool. If you encounter any issues or have any questions when using PACE, please email PLOS at figures@plos.org. Please note that Supporting Information files do not need this step.

# Author comment: Author response to Decision Letter 0

**DOI:** 10.1371/journal.pone.0292049.r002

29 May 2023

Answers to Reviewer 1

1. From the current manuscript, it is hard to understand the intuition why topology is beneficial. Furthermore, clarity of the manuscript can be improved.

The structure of the manuscript has been changed by switching the ordering of subsections in the Materials and Methods section, and many paragraphs have been rewritten. We hope that this revised version meets the criteria and standards of the journal. Topological features of data are the basis of the classifier proposed in this article. It is now more clearly explained in the Topological Classifier subsection that our underlying assumption is that point clouds sampled from different brain states exhibit recognizably different shapes. The plausibility of this claim has been tested by means of bootstrapping on each motivational state. The resulting distributions of zero-dimensional total persistence are significantly different, as now shown in Fig. 3 and ascertained with a Kolmogorov–Smirnov test with p-values below 0.0001. Further evidence of the difference between topological descriptors of the three motivational states is given by the silhouettes depicted in Fig. 4.

2. After PCA, 2-10 dimensional vectors are given. Then, how do you compute the landscape? What corresponds to the “point clouds” in TDA?

Persistence landscapes are computed from persistence diagrams of Vietoris–Rips simplicial complexes calculated from point clouds equipped with Euclidean distance. Point clouds represent vectors extracted from the dataset. The dimension of each point cloud is the dimension of the ambient space, i.e., the number of channels —electrodes or brain sources—, and there is a point for each of 12 blocks of 108 trials per participant. After PCA reduction, the dimension of the ambient space decreases accordingly. Thus, Vietoris–Rips simplicial complexes are computed anew using vectors of the corresponding reduced dimension, and landscapes are then derived from the resulting persistence diagrams.

3. Post processing is still ambiguous to me. The data length of the band spectra or time series is probably not explicitly given. (The width of time bins are not specified.) That dimension size should give a nice intuition if the classification works or not. I would like to see a figure for point clouds, for example. 

The dimension size of the EEG dataset is defined by the number of electrodes placed on the headcap (60). Each electrode records brain activity generated across the brain, but perceivable through one small area on the participant’s skull, where the electrode is placed. As described in the original neuroscience study (Cos et al. 2021), the signals were sampled at 1 KHz at the time of recording during an interval of 1200 ms. The choice of this interval responds to a combination of technical and scientific constraints. First, EEG activity is heavily corrupted by movement artifacts as soon as the movement response is initiated (and sometimes prepared) to the extent that its analysis becomes impossible. Second, the purpose of the original neuroscience study was to observe baseline modulations of social pressure on the participant’s brain state, which could only be reliably assessed prior to each trial presentation of options. This is fully explained in Cos et al. 2021. That all being said, the dataset provided for the present article had been time averaged after independent component analysis and bandpass filter processing. Hence, there is no temporal dimension to consider, but only the number of electrodes (60), the number of trials (432), and the number of brain states (3). Two point clouds, corresponding to participants 1 and 8 in the study after PCA reduction to dimension 3, are now shown in Fig. 11. The shape distinction between the three motivational states M0, M1, M2 can be clearly seen in this image.

4. Each figure is dirty (Figure 3 and later). The labels are too small to read. No label is given on the x- and y- axes in Figure 3. (Units are not available.)

We have rebuilt all the images to achieve higher resolution. Furthermore, we have increased the font sizes of labels and units in the figures, and ensured that units are specified in all diagram axes where units are pertinent. The case of Fig. 3 is special since units in the axes of persistence silhouettes do not have any standard interpretation. To remedy this as much as possible, we have specified the meaning of axes in the caption of Fig. 3, as follows: The x-axis represents lifetime lengths of connected components of the Vietoris–Rips complex, normalized into 1000 vector units by equating ranges. The y-axis represents density of points in the persistence diagram weighted by their lifetime, uniformized by equating height in the pictures.

Answers to Reviewer 2

1. The information to reproduce the results from the manuscript is lacking, and there are also leaps in the discussion. Therefore, it is considered necessary to substantially revise the manuscript about these points.

The dataset used in this study has been uploaded into Kaggle and our code has been uploaded to a GitHub repository. The URLs are indicated below and in page 15 of the manuscript. The Discussion has been rewritten with substantial changes, striving for more clarity and avoiding speculative claims not fully supported by numerical results in the article. Other parts of the draft have also been rewritten; further details are now given in the Materials and Methods section, and two new figures have been added (Fig. 3 and Fig. 11).

2. [lines 28 - 37] Only [27] and [28] are mentioned as previous studies of classification methods using TDA, but there are likely other methods. For example, I found the following ones just by doing a short search: [Genki Kusano, Yasuaki Hiraoka, Kenji Fukumizu Proceedings of The 33rd International Conference on Machine Learning, PMLR 48:2004-2013, 2016.] A more elaborate survey of previous studies should be conducted and the differences between them and the present method should be clearly explained.

The article by Kusano–Hiraoka–Fukumizu about a persistence weighted Gaussian kernel for topological data analysis has been added to the References of the article and is now mentioned in line 27 of the Introduction, together with articles by Bubenik and Bubenik–Dlotko about persistence landscapes. These articles contain indeed important examples of topological descriptors that have proved to be powerful tools for statistical analyses. In the article by Kusano–Hiraoka– Fukumizu, persistence weighted Gaussian kernels are used to achieve an improved accuracy in a support vector machine classifier. The classifier described in our article does not require the use of support vector machines nor of any other machine learning classifier, but it relies on topological descriptors from data. As far as we know, ours is the first article in which a classifier based solely on topological data analysis has been used with experimental data. A precedent that has to be mentioned is the article by Kindelan–Frías–Cerda–Hitschfeld (2021), where Vietoris–Rips simplicial complexes computed from data clouds were used as a refinement of nearest neighbour classifiers, which is very distinct from our method. Our classifier is similar to the one used in Inés–Domínguez–Heras–Mata–Rubio (2023) for semi-supervised machine learning tasks. The purpose of their article is different from ours in that they do not use persistence silhouettes as topological descriptors, and the aim of their work is not related to ours.

3. The reproducibility cannot be checked because the values of all hyperparameters of the methods used in the analysis nor the methods used to determine them are not described in the manuscript. For example, the criteria for noisy channels [line

248], the shape of the window function of the bandpass filter [line 258], and the hyperparameters for “kernel-smoothed” [line 307] should be described. Also, it is highly recommended that the code and data used in the manuscript should be made

publicly available to confirm the reproducibility.

The filter used to band-pass the original temporal series was a fourth-order Butterworth filter with boundary frequencies 8 − 15 Hz for the alpha band, 15 − 32 Hz for the beta band, and 32−80 Hz for the gamma band. Details have been added to our account of the signal processing (lines 114–122 and 277–278). Kernel-smoothing was not used in our analyses; it is only mentioned in order to ease interpretation of violins depicted in Fig. 6 and subsequently. Default parameters from the seaborn library were used to draw violins. That being said, we note that time series for this study were provided already averaged in the dataset. We have not performed any bandpass filtering nor any smoothing when carrying out analyses in the article under consideration. Although the focus of our study is the TDA classifier, we considered appropriate to provide an account of the data-conditioning process for completeness. The full specifics of the original data conditioning are available in a previous neuroscience study (Cos et al. 2021). In order to ensure that data used in the manuscript be publicly available for reproducibility purposes, the dataset has been uploaded to Kaggle, https://www.kaggle.com/ds/3333171, and our code has been uploaded to a public GitHub repository: https://github.com/ainafem/brain_states_motivation.

4. The claims that the proposed method captures geometric structure rather than data variance in general data seem inappropriate as claims deduced from the results of numerical experiments. Therefore, numerical experiments or theoretical analysis supporting such claims, or the withdrawal of the claims, would be necessary for the publication of the manuscript. 

It is true that the concept of “explained shape” as opposed to explained variance that we mentioned in the previous version of the article was not defined in a precise way and was speculative. Therefore we have removed every reference to explained shape from the manuscript. However, the concept of shape of a point cloud is defined rigorously in the framework of topological data analysis —namely, it refers to persistent homology of Vietoris–Rips complexes associated with a given finite set of points in Euclidean space. The classifier that we use in this article is based on comparison of silhouettes drawn from persistence diagrams of Vietoris–Rips complexes and is consequently strongly based on topological invariants of such complexes. In other words, the outcome of our classifier does not directly depend on the variance of the given datasets, but it is derived instead from topological descriptors of data. Furthermore, our results in the article (specifically, Figures 9, 12 and 13) demonstrate that the accuracy of our classifier remains stable in most cases along a range of dimensions for which variance changes steadily (as implicit in principal component analysis). We have rewritten the Results section and the Discussion in order to describe this fact more clearly. In addition, we have added Fig. 3, showing that shape descriptors computed from Vietoris–Rips simplicial complexes are significantly different in the three motivational states studied in the article. This justifies an a priori plausibility of the effectiveness of a classifier based on shape differences between such motivated brain states.

5. [line 59 and 62] Why exclude the cases d = 1 and k = 0 even though 0-dimensional oles are analyzed as described in line 117?

Although the case d = 1 is usually omitted in topological data analysis of point clouds, it is true that all definitions make sense for d = 1 and we have therefore included the case d = 1 in our presentation of persistence descriptors (in line 156). As for k = 0, the 0-simplices are precisely the vertices of the Vietoris–Rips complex and we have made this more explicit in line 159.

6. [line 63] It would be good to have an explanation as to why there are 2 in 2t.

The 2t value is usually chosen for the sake of consistency with the simplicial set Ct(X) obtained from the Cech filtration in which a k-simplex with vertices v0, . . . , vk occurs when closed balls of radius t centered at v0, . . . , vk have a common intersection (since this happens when the distance between their centres is lower than 2t). However, Cech filtrations are not used in our article, and for our purposes it is indistinct to use t or 2t. Therefore, we have now written ∥vi − vj∥ ≤ t for simplicity in line 160 and adjusted persistence values accordingly.

7. [line 67] I think an explanation of the definition of F11 is needed. If it means a finite body of degree 2, is it not more common to use F2?

The notation F11 was meant to denote a finite field of 11 elements. While this choice of 11 may seem peculiar, it is the default choice used by the GUDHI software. This choice is plausible since 1 is different from −1 for odd primes (which would be relevant if torsion phenomena were meaningful for analysis), and the prime number p = 11 is allegedly well suited in geometric or number-theoretic contexts.

8. [line 149 and others] The definition of word “shape” is unclear. It would be easier for the reader to consider the effectiveness of the algorithm if it were made explicit about what structure is assumed.

This part of the Materials and Methods section has been rewritten, and Fig. 3 has been added to illustrate the meaning of shape distinctions between brain states within the data cloud. Indeed, “shape” is quantified by descriptors from persistence diagrams, such as total persistence, landscapes or silhouettes.

9. [Fig. 4] An explanation of the abbreviation Mot. St. was not found anywhere in the manuscript and should be mentioned somewhere. 

The abbreviation “Mot. St.” appears in Fig. 5. The meaning of all abbreviations used in Fig. 5 has been specified in the caption.

10. [Fig. 5] It should explain why it is classified on a per-subject basis.

The dataset used to test our TDA classifier was recorded in Cos et al. 2021. Their goal was to characterize the motivated brain state of each participant as a function of the manipulation of social pressure. Hence, the original analysis was made on a single-participant basis. The interest in making single-participant analysis is bound to two main reasons: First, it is due to the fact that each recording stems from a different brain, with significant differences in substrate. Second, with an eye on the possibility of using these techniques with clinical patients, it was of significant relevance to show that analyses were successful on single-participant data.

11. [lines 309-316] The explanation of this paragraph was unclear to me so it would improve readability if it were rewritten for clarity.

This paragraph has been rewritten and it now reads as follows, hopefully more clearly: “Using this as a baseline, we assessed the influence of dimensionality reduction on classification accuracy. For this, we applied two complementary dimensionality 

reduction techniques. For each participant dataset, we first performed a principal component analysis (PCA) decomposition, selecting the dimensions that would explain until 95% of data variance for all participants, ultimately retaining from 2 to 10 dimensions.”

Attachment Submitted filename: Response_to_Reviewers.pdf Click here for additional data file.

# Peer review: Decision Letter 1

**DOI:** 10.1371/journal.pone.0292049.r003

**Authors:** Ruriko Yoshida

13 Jun 2023

PONE-D-23-06017R1A topological classifier to characterize brain states: When shape matters more than variancePLOS ONE

Dear Dr. Ferrà,

Thank you for submitting your manuscript to PLOS ONE. After careful consideration, we feel that it has merit but does not fully meet PLOS ONE’s publication criteria as it currently stands. Therefore, we invite you to submit a revised version of the manuscript that addresses the points raised during the review process.

Even though Reviewer 1 is happy with your edits, Reviewer 2 gave comments that he needs to see what you edited clearly. So please indicate clearly what you modified and what you deleted and send it back to us. 

Please submit your revised manuscript by Jul 28 2023 11:59PM. If you will need more time than this to complete your revisions, please reply to this message or contact the journal office at plosone@plos.org. When you're ready to submit your revision, log on to https://www.editorialmanager.com/pone/ and select the 'Submissions Needing Revision' folder to locate your manuscript file.

Please include the following items when submitting your revised manuscript:

- A rebuttal letter that responds to each point raised by the academic editor and reviewer(s). You should upload this letter as a separate file labeled 'Response to Reviewers'.
- A marked-up copy of your manuscript that highlights changes made to the original version. You should upload this as a separate file labeled 'Revised Manuscript with Track Changes'.
- An unmarked version of your revised paper without tracked changes. You should upload this as a separate file labeled 'Manuscript'.

If you would like to make changes to your financial disclosure, please include your updated statement in your cover letter. Guidelines for resubmitting your figure files are available below the reviewer comments at the end of this letter.

If applicable, we recommend that you deposit your laboratory protocols in protocols.io to enhance the reproducibility of your results. Protocols.io assigns your protocol its own identifier (DOI) so that it can be cited independently in the future. For instructions see: https://journals.plos.org/plosone/s/submission-guidelines#loc-laboratory-protocols. Additionally, PLOS ONE offers an option for publishing peer-reviewed Lab Protocol articles, which describe protocols hosted on protocols.io. Read more information on sharing protocols at https://plos.org/protocols?utm_medium=editorial-email&utm_source=authorletters&utm_campaign=protocols.

We look forward to receiving your revised manuscript.

Kind regards,

Ruriko Yoshida

Academic Editor

PLOS ONE

Additional Editor Comments:

Even though Reviewer 1 is happy with your edits, Reviewer 2 gave comments that he needs to see what you edited clearly. So please indicate clearly what you modified and what you deleted and send it back to us.

[Note: HTML markup is below. Please do not edit.]

Reviewers' comments:

Reviewer's Responses to Questions

**Comments to the Author**

1. If the authors have adequately addressed your comments raised in a previous round of review and you feel that this manuscript is now acceptable for publication, you may indicate that here to bypass the “Comments to the Author” section, enter your conflict of interest statement in the “Confidential to Editor” section, and submit your "Accept" recommendation.

Reviewer #1: All comments have been addressed

Reviewer #2: (No Response)

**********

2. Is the manuscript technically sound, and do the data support the conclusions?

The manuscript must describe a technically sound piece of scientific research with data that supports the conclusions. Experiments must have been conducted rigorously, with appropriate controls, replication, and sample sizes. The conclusions must be drawn appropriately based on the data presented. 

Reviewer #1: Yes

Reviewer #2: (No Response)

**********

3. Has the statistical analysis been performed appropriately and rigorously? 

Reviewer #1: Yes

Reviewer #2: (No Response)

**********

4. Have the authors made all data underlying the findings in their manuscript fully available?

The PLOS Data policy requires authors to make all data underlying the findings described in their manuscript fully available without restriction, with rare exception (please refer to the Data Availability Statement in the manuscript PDF file). The data should be provided as part of the manuscript or its supporting information, or deposited to a public repository. For example, in addition to summary statistics, the data points behind means, medians and variance measures should be available. If there are restrictions on publicly sharing data—e.g. participant privacy or use of data from a third party—those must be specified.

Reviewer #1: Yes

Reviewer #2: (No Response)

**********

5. Is the manuscript presented in an intelligible fashion and written in standard English?

PLOS ONE does not copyedit accepted manuscripts, so the language in submitted articles must be clear, correct, and unambiguous. Any typographical or grammatical errors should be corrected at revision, so please note any specific errors here.

Reviewer #1: Yes

Reviewer #2: (No Response)

**********

6. Review Comments to the Author

Please use the space provided to explain your answers to the questions above. You may also include additional comments for the author, including concerns about dual publication, research ethics, or publication ethics. (Please upload your review as an attachment if it exceeds 20,000 characters)

Reviewer #1: The authors appropriately responded to the questions and comments which I raised in the previous turn.

Reviewer #2: Please send me a manuscript that shows the diff from the previous manuscript.

Specifically, I would like to know what you have deleted, what you have added, and what you have modified.

**********

7. PLOS authors have the option to publish the peer review history of their article (what does this mean?). If published, this will include your full peer review and any attached files.

If you choose “no”, your identity will remain anonymous but your review may still be made public.

**Do you want your identity to be public for this peer review?** For information about this choice, including consent withdrawal, please see our Privacy Policy.

Reviewer #1: No

Reviewer #2: No

**********

[NOTE: If reviewer comments were submitted as an attachment file, they will be attached to this email and accessible via the submission site. Please log into your account, locate the manuscript record, and check for the action link "View Attachments". If this link does not appear, there are no attachment files.]

While revising your submission, please upload your figure files to the Preflight Analysis and Conversion Engine (PACE) digital diagnostic tool, https://pacev2.apexcovantage.com/. PACE helps ensure that figures meet PLOS requirements. To use PACE, you must first register as a user. Registration is free. Then, login and navigate to the UPLOAD tab, where you will find detailed instructions on how to use the tool. If you encounter any issues or have any questions when using PACE, please email PLOS at figures@plos.org. Please note that Supporting Information files do not need this step.

# Author comment: Author response to Decision Letter 1

**DOI:** 10.1371/journal.pone.0292049.r004

27 Jun 2023

At your request, a new version of the "Revised Manuscript with Track Changes" file has been uploaded to the server. In this file, the changes made in the original manuscript submitted on March 1 have been highlighted more clearly than in our previous revision with track changes dated May 20. In the current version, deleted text is crossed out and marked in red, while added text is marked in blue. In addition, a few comments are marked in brown.

Attachment Submitted filename: response_to_reviewers.pdf Click here for additional data file.

# Peer review: Decision Letter 2

**DOI:** 10.1371/journal.pone.0292049.r005

**Authors:** Ruriko Yoshida

12 Sep 2023

A topological classifier to characterize brain states: When shape matters more than variance

PONE-D-23-06017R2

Dear Dr. Ferrà,

We’re pleased to inform you that your manuscript has been judged scientifically suitable for publication and will be formally accepted for publication once it meets all outstanding technical requirements.

Within one week, you’ll receive an e-mail detailing the required amendments. When these have been addressed, you’ll receive a formal acceptance letter and your manuscript will be scheduled for publication.

An invoice for payment will follow shortly after the formal acceptance. To ensure an efficient process, please log into Editorial Manager at http://www.editorialmanager.com/pone/, click the 'Update My Information' link at the top of the page, and double check that your user information is up-to-date. If you have any billing related questions, please contact our Author Billing department directly at authorbilling@plos.org.

If your institution or institutions have a press office, please notify them about your upcoming paper to help maximize its impact. If they’ll be preparing press materials, please inform our press team as soon as possible -- no later than 48 hours after receiving the formal acceptance. Your manuscript will remain under strict press embargo until 2 pm Eastern Time on the date of publication. For more information, please contact onepress@plos.org.

Kind regards,

Ruriko Yoshida

Academic Editor

PLOS ONE

Reviewers' comments:

Reviewer's Responses to Questions

**Comments to the Author**

1. If the authors have adequately addressed your comments raised in a previous round of review and you feel that this manuscript is now acceptable for publication, you may indicate that here to bypass the “Comments to the Author” section, enter your conflict of interest statement in the “Confidential to Editor” section, and submit your "Accept" recommendation.

Reviewer #1: All comments have been addressed

**********

2. Is the manuscript technically sound, and do the data support the conclusions?

The manuscript must describe a technically sound piece of scientific research with data that supports the conclusions. Experiments must have been conducted rigorously, with appropriate controls, replication, and sample sizes. The conclusions must be drawn appropriately based on the data presented. 

Reviewer #1: Yes

**********

3. Has the statistical analysis been performed appropriately and rigorously? 

Reviewer #1: Yes

**********

4. Have the authors made all data underlying the findings in their manuscript fully available?

The PLOS Data policy requires authors to make all data underlying the findings described in their manuscript fully available without restriction, with rare exception (please refer to the Data Availability Statement in the manuscript PDF file). The data should be provided as part of the manuscript or its supporting information, or deposited to a public repository. For example, in addition to summary statistics, the data points behind means, medians and variance measures should be available. If there are restrictions on publicly sharing data—e.g. participant privacy or use of data from a third party—those must be specified.

Reviewer #1: Yes

**********

5. Is the manuscript presented in an intelligible fashion and written in standard English?

PLOS ONE does not copyedit accepted manuscripts, so the language in submitted articles must be clear, correct, and unambiguous. Any typographical or grammatical errors should be corrected at revision, so please note any specific errors here.

Reviewer #1: Yes

**********

6. Review Comments to the Author

Please use the space provided to explain your answers to the questions above. You may also include additional comments for the author, including concerns about dual publication, research ethics, or publication ethics. (Please upload your review as an attachment if it exceeds 20,000 characters)

Reviewer #1: The authors appropriately responded to the questions and comments which I raised in the previous turn.

**********

7. PLOS authors have the option to publish the peer review history of their article (what does this mean?). If published, this will include your full peer review and any attached files.

If you choose “no”, your identity will remain anonymous but your review may still be made public.

**Do you want your identity to be public for this peer review?** For information about this choice, including consent withdrawal, please see our Privacy Policy.

Reviewer #1: No

**********

# Editor report: Acceptance letter

**DOI:** 10.1371/journal.pone.0292049.r006

**Authors:** Ruriko Yoshida

21 Sep 2023

PONE-D-23-06017R2 

A topological classifier to characterize brain states: When shape matters more than variance 

Dear Dr. Ferrà:

I'm pleased to inform you that your manuscript has been deemed suitable for publication in PLOS ONE. Congratulations! Your manuscript is now with our production department. 

If your institution or institutions have a press office, please let them know about your upcoming paper now to help maximize its impact. If they'll be preparing press materials, please inform our press team within the next 48 hours. Your manuscript will remain under strict press embargo until 2 pm Eastern Time on the date of publication. For more information please contact onepress@plos.org.

If we can help with anything else, please email us at plosone@plos.org. 

Thank you for submitting your work to PLOS ONE and supporting open access. 

Kind regards, 

PLOS ONE Editorial Office Staff

on behalf of

Dr. Ruriko Yoshida 

Academic Editor

PLOS ONE
